# Conducting Polymers for Electrochemical Sensing: From Materials and Metrology to Intelligent and Sustainable Biointerfaces

**DOI:** 10.3390/s26030908

**Published:** 2026-01-30

**Authors:** Giovanna Di Pasquale, Antonino Pollicino

**Affiliations:** 1 Department of Chemical Sciences, University of Catania, V.le A. Doria 6, 95125 Catania, Italy; 2 Department of Civil Engineering and Architecture, University of Catania, V.le A. Doria 6, 95125 Catania, Italy

**Keywords:** conducting polymers, organic mixed ionic–electronic conductors (OMIECs), electrochemical biosensors, biointerfaces, wearable and implantable sensors, drift and fouling, metrology and calibration, nanostructured polymer films, transient and biodegradable electronics, data-driven materials design

## Abstract

**Highlights:**

**What are the main findings?**

- Reported performance metrics of conducting polymer–based electrochemical biosensors (LOD, sensitivity, linear range, stability) are strongly affected by metrological variability arising from polymer formulation, film morphology, electrode geometry, electrolyte composition, and measurement protocols.
- A critical analysis reveals that the apparent performance gains reported across PEDOT:PSS, polyaniline, polypyrrole, and polythiophenes are often not directly comparable due to non-standardized calibration procedures, reference electrode instability, and inconsistent data treatment.

**What are the implications of the main findings?**

- The lack of standardized metrology represents a primary barrier to reproducibility, long-term reliability, and technology transfer of conducting polymer–based biosensors from laboratory demonstrations to real-world applications.
- The adoption of unified reporting frameworks—including explicit uncertainty analysis, statistically validated LOD definitions, and protocol-level transparency—is essential for meaningful benchmarking and rational device optimization.

**Abstract:**

Conducting polymers (CPs) have become cornerstone materials in electrochemical sensors and biosensors due to their mixed ionic–electronic conduction, mechanical softness, and intrinsic biointerface compatibility. This review provides a comprehensive and critical overview of the field, tracing the evolution of CP-based devices from classical poly(3,4-ethylenedioxythiophene):poly(styrene sulfonate) (PEDOT:PSS), polyaniline (PANI), and polypyrrole (PPy) electrodes to emerging nanostructured, hybrid, wearable, and transient systems. We discuss fundamental charge-transport mechanisms, doping strategies, structure–property relationships, and the role of morphology and biofunctionalization in dictating sensitivity, selectivity, and stability. Particular emphasis is placed on reliability challenges—including drift, dopant leaching, environmental degradation, and biofouling—and on the current lack of standardized metrology, which hampers cross-study comparability. We propose a framework for rigorous calibration, reference electrode design, and data reporting, enabling quantitative benchmarking across materials and architectures. To support meaningful cross-platform comparison, representative performance envelopes—including conductivity, limit of detection, sensitivity, selectivity strategies, and operational stability—are critically benchmarked across major CP families and sensing modalities. Finally, we explore future directions such as organic mixed ionic–electronic conductors, biohybrid and living polymer interfaces, Artificial Intelligence-driven modeling, and sustainable transient electronics.

## 1. Introduction

Electrochemical sensors and biosensors have become indispensable analytical tools for environmental monitoring, clinical diagnostics, and wearable health assessment. Their impact derives from the ability to transduce biochemical events—typically redox or ion-exchange processes—into measurable electrical signals with high sensitivity, selectivity, and operational stability. CPs play a central role in this context, providing a unique combination of electronic conductivity, ionic permeability, and mechanical softness that is rarely matched by inorganic or carbon-based materials. Since the pioneering works on PPy, PANI, and PEDOT:PSS in the 1980s [1,2,3], CPs have evolved from simple conductive coatings into multifunctional, biointeractive interfaces capable of supporting direct electron/ion exchange with complex chemical and biological environments. While PEDOT:PSS remains the most pervasive CP due to its aqueous processability, tunable mixed conduction, and excellent biocompatibility, the landscape is rapidly expanding. Recent advances in Organic mixed ionic–electronic conductors (OMIECs)—including PEDOT:Tos, P3CPT, and side-chain-engineered thiophenes—demonstrate improved volumetric capacitance, enhanced chemical stability in physiological conditions, and reduced reliance on polyelectrolyte dopants. These materials broaden the palette of available CP systems and point toward a new generation of tailored transducing layers for bioelectronics.

The appeal of CPs lies in their intrinsically hybrid nature. Their π-conjugated backbone supports efficient charge delocalization, while the presence of mobile dopants enables ion exchange with the surrounding medium [4,5]. Such dual transport allows CPs to couple intimately with hydrated biological environments, dampen mechanical mismatch, and support the immobilization of biomolecules through electrostatic, covalent, or affinity interactions [6]. As a result, they serve as “soft conductors” that bridge the rigid inorganic world of metals and carbon nanomaterials with the dynamic, hydrated complexity of tissues and biofluids [5,7].

Over the past decade, significant progress in materials chemistry and processing has transformed the capabilities of CP-based electrodes. Nanostructured morphologies—such as nanotubes, nanofibers, porous films, and hydrogels—have greatly increased electroactive surface area and improved analyte diffusion [8]. Hybrid architectures combining CPs with carbon nanomaterials (Carbon Nanotubes (CNTs), graphene, MXenes) or metal nanoparticles have enabled synergistic enhancements in conductivity, catalytic activity, and mechanical resilience [9,10]. More recently, CP hydrogels and bio-composites have paved the way for soft, stretchable, and even transient bioelectronic devices capable of skin-conformal sensing or implantation in dynamic, hydrated environments [11,12].

Despite these advances, substantial challenges remain. Long-term electrical drift, dopant instability, and biofouling at the polymer–electrolyte or polymer–tissue interface can limit reproducibility and device lifetime [13]. Furthermore, the literature often reports inconsistent characterization protocols and performance metrics—particularly regarding limit of detection (LOD), linear range, operational stability, and selectivity—which complicate quantitative comparison across studies [9]. Establishing clear structure–property–performance relationships, supported by rigorous metrological practices, is therefore essential for the rational design and benchmarking of CP-based (bio)sensors. Several recent reviews have comprehensively addressed specific aspects of conducting polymer-based electrochemical sensors, including materials chemistry, device architectures, or selected application domains. However, many of these contributions primarily emphasize descriptive comparisons or record performance metrics, often without systematically addressing long-term reliability, inter-study comparability, and metrological consistency. In contrast, the present review explicitly integrates materials design with degradation physics, standardized performance metrics, and benchmarking strategies, with the aim of identifying unresolved challenges and practical trade-offs that limit real-world deployment.

### Unresolved Challenges and Open Questions

Despite major progress in materials engineering and device demonstrations, several unresolved challenges—already highlighted but often insufficiently addressed in recent review articles—continue to limit the maturity and comparability of CP-based electrochemical (bio)sensors. First, long-term drift remains poorly decomposed into its coupled contributors (dopant migration/leaching, interfacial water layers, swelling, overoxidation, and biofouling), making lifetime prediction difficult. Second, inter-batch and inter-laboratory reproducibility is still rarely quantified beyond small sample sizes, hampering reliable calibration transfer. Third, direct benchmarking across polymer families and sensing modalities is often ambiguous due to inconsistent definitions of LOD, normalization choices, and testing matrices. Finally, wearable and implantable deployment highlights additional constraints—reference electrode stability, encapsulation, mechanical fatigue, and regulatory-grade validation—that are frequently under-addressed in laboratory reports. This review addresses these gaps by integrating structure–property design with reliability physics and metrological standardization, providing quantitative benchmarking tools and actionable design rules.

We survey the latest developments in CPs for electrochemical sensing and biosensing, emphasizing:

(i) materials chemistry and emerging OMIECs;
(ii) structure–property relationships governing charge transport and transduction;
(iii) engineering strategies for enhancing stability, reproducibility, and antifouling; and
(iv) new directions in wearable, implantable, and sustainable CP-based bioelectronics.

By explicitly reframing these recurring issues as coupled materials–metrology problems rather than isolated device limitations, this review aims to move beyond descriptive summaries and provide a critical framework for evaluating progress and guiding future research.

## 2. Fundamentals of Conducting Polymers for Electrochemical Sensing

**Figure 1:**
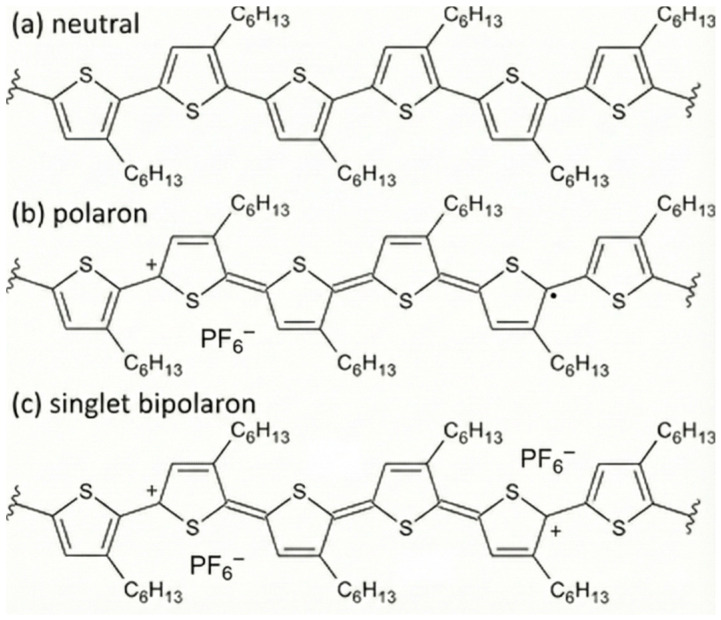
Schematic representation of the resonance structures of poly(3-hexylthiophene) (P3HT) in different oxidation states. (**a**) Polymer chain in the neutral state exhibiting a benzenoid (aromatic) structure. (**b**) Formation of a polaron (radical cation) following partial oxidation, showing a rearrangement to the quinoid structure and the presence of the counterion. (**c**) Formation of a singlet bipolaron (dication) stabilized by two counterions.

CPs derive their remarkable properties from a π-conjugated backbone capable of supporting delocalized charge carriers. Unlike insulating polymers, which possess localized σ-bonds, CPs exhibit extended π-electron delocalization along the chain, enabling the formation of polarons and bipolarons—localized quasi-particles responsible for charge transport [14] (Figure 1). When chemically or electrochemically doped, these conjugated systems transition from semiconducting to metallic-like behavior, with conductivities spanning from 10^−5^ to >10^3^ S cm^−1^ depending on the degree of doping, structural order, and dopant mobility [15].

### 2.1. Doping and Mixed Conduction

**Figure 2:**
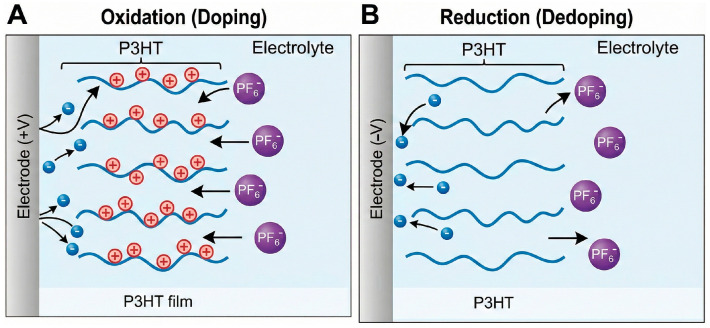
Schematic representation of electron-ion coupling in P3HT during redox cycling. (**A**) Upon oxidation electrons are removed, and anions from the electrolyte insert into the film to maintain charge neutrality. (**B**) Upon reduction, electrons are injected, and anions are expelled into the electrolyte.

The cornerstone of CP functionality lies in doping/dedoping processes, where electronic charge transfer is compensated by the ingress or egress of ions from the electrolyte. During oxidation (“p-doping”), the polymer backbone acquires positive charges balanced by anions, whereas reduction (“n-doping”) introduces negative charges balanced by cations. Importantly, the identity, mobility, and binding strength of these counter-ions can be deliberately tuned through dopant chemistry, polymer structure, and electrochemical conditions, enabling CPs to operate as selective anion or cation exchangers depending on the sensing architecture. This interplay of electron and ion motion endows CPs with a mixed ion–electron conduction mechanism, absent in conventional metals or carbon materials [4]. Such dual conductivity allows CPs to interface efficiently with ionic media—including biological fluids—and to transduce redox or ionic changes into measurable electronic signals [16] (Figure 2).

The ionic component also imparts volumetric capacitance, a property that underpins the widespread use of CPs in electrochemical capacitors and selected sensing architectures. For example, mixed ionic–electronic conducting PVA/PEDOT:PSS hydrogels have been shown to exhibit volumetric capacitance values on the order of several F·cm^−3^ in organic electrochemical transistor channels, evidencing ionic charge storage throughout the bulk material rather than being confined to the electrode–electrolyte interface [17]. High-capacitance all-polymer hydrogels based on PEDOT networks have similarly demonstrated significant charge storage capacity suitable for flexible and conformable electrodes [18]. More generally, CP composites have been explored as active masses in supercapacitor electrodes, highlighting the combined contribution of pseudocapacitive redox processes and double-layer effects.

Importantly, in contrast to purely interfacial double-layer capacitance, CPs exhibit volumetric, redox-assisted charge storage, in which ionic compensation accompanies electronic charging throughout the polymer bulk. This mechanism reduces interfacial impedance and enhances transduction efficiency, rather than generating parasitic capacitive currents. While excessive capacitive contributions are generally undesirable in conventional amperometric or voltammetric sensing—where they may obscure faradaic signals—controlled bulk capacitance becomes advantageous in impedance-based sensors and organic electrochemical transistor architectures, where it stabilizes the electrochemical response and improves signal-to-noise ratios in hydrated or soft environments [19]. This behavior has been particularly well documented for PEDOT:PSS, whose polyelectrolyte counterion (PSS^−^) preserves electronic percolation while enabling reversible ionic exchange within the film [20]. In biosensing contexts, such volumetric ion accessibility facilitates real-time monitoring of ion fluxes, metabolite concentrations, and enzymatic reactions in aqueous matrices.

### 2.2. Structure–Property Relationships

The molecular structure, doping level, and morphology of CPs critically determine their electrical and electrochemical behavior. Ordered domains (crystalline lamellae or π–π stacks) enhance carrier mobility, while amorphous regions promote ion penetration and mechanical compliance [21]. Optimizing the balance between these two is essential: excessive ordering can limit ion diffusion, whereas excessive swelling can lead to drift and structural fatigue. The incorporation of secondary dopants such as ethylene glycol (EG), dimethyl sulfoxide (DMSO), or ionic liquids (ILs) reorganizes the microstructure of PEDOT:PSS, increasing conductivity by several orders of magnitude while modulating hydrophilicity and adhesion [22]. Similarly, protonic acids and surfactants are used to tune the oxidation state and conductivity of PANI or PPy films. In PANI, protonic acids induce reversible protonation of imine nitrogen sites, stabilizing polaronic charge carriers without changing the electron count of the backbone. In PPy, surfactant dopants act both as charge-compensating anions and as structure-directing agents, influencing film porosity, ion mobility, and mechanical compliance.

### 2.3. Electrochemical Behavior and Charge Transport

At the electrode–electrolyte interface, CPs act as dynamic redox-active layers whose transport properties are governed by electrochemical doping and dedoping processes. Upon oxidation (p-doping), positive charges are generated along the conjugated backbone and compensated by anion ingress from the electrolyte, whereas reduction (n-doping) involves cation uptake. This coupled motion of electronic and ionic species gives rise to a mixed ion–electron conduction mechanism that is central to the operation of CP-based electrochemical sensors.

In their doped state, CPs exhibit electrical conductivities spanning several orders of magnitude, depending on polymer chemistry, structural order, and doping strategy. Moderately ordered polypyrrole or polyaniline films typically display conductivities of ~10^−1^–10^1^ S·cm^−1^, whereas highly optimized PEDOT-based systems—such as PEDOT:PSS with secondary dopants or hydrated PEDOT networks—can reach values approaching 10^2^–10^3^ S·cm^−1^. Recent experimental studies on mixed ionic–electronic PEDOT:PSS hydrogels have demonstrated stable electronic conduction combined with efficient ionic transport, confirming their suitability for electrochemical operation in aqueous and biological environments [17].

Under small potential excursions around the operating potential, CPs behave as linear mixed conductors, with charge transport dominated by capacitive charging of the polymer bulk coupled to ion diffusion within the hydrated matrix. In this regime, electrochemical impedance spectroscopy (EIS) typically reveals low charge-transfer resistance values (R_ct_ on the order of tens to a few hundreds of Ω·cm^2^) and a pronounced low-frequency capacitive response, reflecting facile ionic penetration and a percolating electronic network. Such behavior has been experimentally observed in PEDOT:PSS-based conductive layers and hydrogels, where the impedance response is strongly modulated by hydration level and doping state [17,23].

At higher polarization potentials, faradaic processes associated with redox transitions of the polymer backbone become dominant and are readily captured by cyclic voltammetry (CV). PEDOT-based materials typically exhibit broad and reversible redox waves within a potential window of approximately −0.2 to +0.8 V vs. Ag/AgCl. The linear dependence of peak current on scan rate observed in these systems is indicative of volumetric, rather than surface-confined, charge storage, consistent with mixed ionic–electronic transport [17,23].

Overall, effective charge transport in CPs arises from the interplay between electronic pathways—governed by hopping between localized states and band-like transport in ordered domains—and ionic diffusion through the polymer network. In electrochemical sensing, this interpenetration of electronic and ionic pathways directly controls both sensitivity and temporal response, enabling rapid equilibration while preserving signal reproducibility over multiple redox cycles.

### 2.4. Interface Chemistry and Biofunctionalization

Beyond their intrinsic conductivity, CPs are chemically versatile materials whose interfacial chemistry can be deliberately engineered to enable specific biofunctionalization. The presence of heteroatoms within the polymer backbone or side chains (e.g., N in polyaniline, S in PEDOT-based systems) allows post-functionalization with biological ligands, redox mediators, and crosslinking agents, transforming CP films from passive conductors into active biointerfaces for sensing applications.

A wide range of biological recognition elements has been immobilized onto CPs, including enzymes, antibodies, aptamers, and peptides. Enzymatic biofunctionalization remains the most established approach, with oxidoreductases such as glucose oxidase or dehydrogenases immobilized within PEDOT:PSS or PPy matrices to enable amperometric detection. Recent experimental studies have shown that direct enzyme immobilization within PEDOT:PSS preserves catalytic activity while improving signal stability and sensitivity due to intimate electrical coupling between the enzyme and the polymer matrix [24,25]. Similarly, affinity-based immobilization of antibodies on PEDOT:PSS/graphene conductive inks has been demonstrated to significantly enhance selectivity and operational stability in electrochemical immunosensors [26].

Redox mediators are frequently integrated into CP matrices to facilitate charge transfer between bioreceptors and the electrode. Ferrocene- and quinone-based mediators, either covalently tethered to the polymer or physically confined within the CP network, have been shown to lower the operating potential and increase signal-to-noise ratios by accelerating electron-transfer kinetics [27,28]. In these architectures, the CP acts both as a structural scaffold and as a mediator reservoir, improving response reproducibility under repeated cycling.

Crosslinking strategies play a critical role in stabilizing biofunctional CP interfaces. Covalent coupling via carbodiimide (EDC/NHS) chemistry has been widely employed to anchor enzymes or antibodies to carboxyl-functionalized CPs, markedly reducing biomolecule leaching and signal drift during long-term operation [29]. More advanced crosslinked PEDOT-based networks further improve mechanical robustness and electrochemical stability, albeit sometimes at the expense of reduced molecular mobility within the film [30].

**Figure 3:**
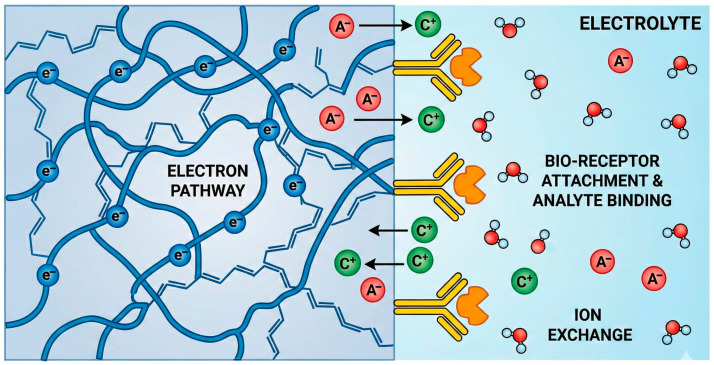
Conceptual illustration of mixed ionic–electronic transduction at a conducting polymer biointerface. The schematic represents experimentally established sensing configurations widely reported in the literature (e.g., impedimetric and transistor-based sensors) and is intended to summarize common transduction principles rather than depict a specific device.

In parallel with chemical tunability, CPs exhibit mechanical properties highly favorable for biointerfacing. PEDOT-based films and hydrogels typically display Young’s moduli in the range of ~10^2^–10^4^ kPa, closely matching those of soft biological tissues. Experimental studies have demonstrated that such mechanical compliance minimizes interfacial stress under deformation, leading to improved electrical stability in wearable and implantable sensors [17,18]. The combined optimization of interface chemistry and mechanical compliance thus enables CP-based sensors to achieve enhanced sensitivity, selectivity, and long-term reliability (Figure 3).

### 2.5. Processing and Patterning Techniques

The processability of CPs is a decisive factor for device fabrication, as processing routes directly determine film morphology, adhesion, electrochemical accessibility, and long-term sensing performance.

Electropolymerization enables conformal coating of complex electrode geometries with precise control over film thickness and oxidation state, while allowing the direct incorporation of dopants, redox mediators, or biomolecules during growth. This approach has been experimentally validated for biosensing, where enzyme entrapment during electropolymerization yields intimate electrical coupling and improved amperometric response. However, limitations include restricted scalability and sensitivity to local current distribution, which can introduce thickness and property inhomogeneities that impact calibration and device-to-device reproducibility. These aspects have been discussed in recent experimental studies on electropolymerized CP-based biosensors and microelectrode arrays, highlighting the trade-off between interfacial precision and large-area uniformity [31,32].

Solution-based deposition techniques (spin-coating, drop-casting, spray-coating) are widely adopted for PEDOT:PSS dispersions owing to their simplicity, low processing temperatures, and compatibility with flexible substrates. Experimental works on PEDOT:PSS and PEDOT:PSS-based composites have shown that such methods enable effective electrochemical sensing in complex media (e.g., artificial sweat), but also revealed limitations related to film thickness control, drying-induced heterogeneity, and ion-transport constraints in thicker layers. These processing-induced effects directly influence impedance response, sensitivity, and operational stability in wearable sensor configurations [23,33].

Vapor-phase polymerization (VPP) produces highly ordered, solvent-free PEDOT films with excellent conductivity and superior batch-to-batch reproducibility. Recent experimental demonstrations of VPP-PEDOT in electrochemical sensing have shown enhanced signal stability and low noise, attributable to the dense yet well-connected polymer morphology. Nonetheless, the relatively harsh processing conditions and limited compatibility with biomolecules restrict the direct integration of biological recognition elements, confining VPP films primarily to transducer layers rather than fully biofunctionalized interfaces [34,35].

Printing and microfabrication techniques, including inkjet printing, aerosol jet printing, and emerging three-dimensional (3D) printing approaches, extend CPs to large-area, patterned, and multiplexed sensor architectures. Comparative experimental studies have shown that printing method and ink formulation strongly affect line continuity, surface roughness, and electrochemical performance, with aerosol jet printing generally offering higher resolution at the expense of tighter process windows. While these techniques enable scalable fabrication of integrated sensor arrays, limitations such as nozzle clogging, variability in feature dimensions, and increased interfacial resistance must be carefully managed to ensure reliable sensing performance [36,37].

Overall, each processing strategy imposes characteristic trade-offs between structural control, scalability, and biointegration. Understanding how processing-induced morphology and interfaces affect ion diffusion, charge-transfer resistance, and mechanical robustness is therefore essential for the rational design of CP-based sensors with optimized sensitivity, reproducibility, and long-term stability.

### 2.6. Key Insights for Sensing Applications

The combination of electronic conductivity, ionic permeability, and chemical functionalizability makes CPs uniquely positioned as transducing materials for electrochemical sensors. Their ability to accommodate both charge carriers and ions within the same matrix allows the direct translation of biochemical reactions—often involving ionic or pH changes—into electrical signals without additional mediators. However, this same responsiveness implies sensitivity to environmental conditions such as pH, ionic strength, and humidity, which must be carefully controlled or compensated in sensor calibration [9]. Thus, understanding the fundamental physicochemical processes governing CP behavior is a prerequisite for reliable device design and for interpreting electrochemical data quantitatively.

## 3. Families of Conducting Polymers and Their Properties

**Figure 4:**
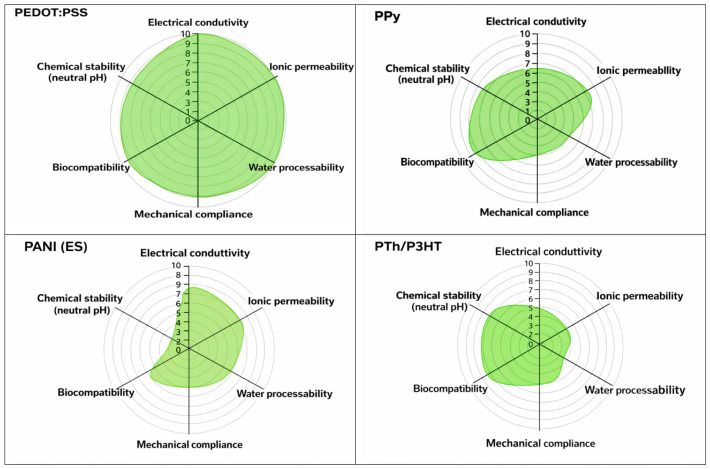
Comparative overview of the properties of the main conducting polymers. The diagrams illustrate the performance profiles of PEDOT:PSS, PPy, PANI (ES), and PTh/P3HT across six critical parameters for bioelectronics. Values on the 0–10 scale represent an arbitrary metric used exclusively for comparison. They are intended to provide a visual representation of general trends and relative strengths, rather than precise absolute measurements.

CPs comprise a chemically diverse class of π-conjugated macromolecules whose electronic, ionic, and interfacial behaviors can be precisely engineered through monomer structure, doping chemistry, and processing. Among the many candidates, four families dominate electrochemical sensing and biosensing research: PEDOT:PSS, PANI, PPy, and polythiophene derivatives (PTh, P3HT, etc.). Each displays a characteristic balance between conductivity, stability, processability, and biocompatibility, which ultimately governs their suitability for specific sensing platforms (Table 1 and Figure 4). Rather than reproducing individual datasets, Figure 4 provides a consolidated visualization of experimentally observed trends reported across multiple primary studies, enabling comparison of relative strengths and limitations among CP families.

### 3.1. Poly(3,4-ethylenedioxythiophene):Poly(styrene sulfonate)

PEDOT:PSS has become the de facto standard among CPs for bioelectronic interfaces thanks to its high conductivity (10^2^–10^3^ S cm^−1^), aqueous processability, and exceptional environmental stability [4,38]. The sulfonate counter-ion (PSS^−^) serves both as dopant and dispersant, allowing the polymer to be cast from water while retaining mixed ionic–electronic conduction. PEDOT:PSS films exhibit low impedance and volumetric capacitance (~40 F cm^−3^), features ideal for electrochemical sensors operating in ionic media.

Beyond foundational studies, a number of recent experimental works have quantitatively demonstrated how processing strategies and additive blending in PEDOT:PSS directly translate into improved electrochemical transduction and operational stability. For instance, textile-integrated PEDOT:PSS organic electrochemical transistors have enabled reliable ion and metabolite sensing in wearable formats under real operating conditions, while composite PEDOT:PSS systems have been shown to balance volumetric capacitance, sensitivity, and long-term signal stability [39,40,41].

Secondary doping with EG, DMSO, or ILs further enhances crystallinity and carrier mobility, while additives such as surfactants or crosslinkers (e.g., (3-glycidyloxypropyl)trimethoxysilane (GOPS)) tune film adhesion and water stability [40,42]. The polymer’s biocompatibility—confirmed in neural implants and skin-mounted electrodes [43]—stems from its hydrophilicity and moderate modulus (~10–100 MPa), which reduce inflammatory responses. Consequently, PEDOT:PSS underpins a wide range of biosensors, from glucose and lactate oxidase electrodes to neural probes and wearable sweat sensors [44].

### 3.2. Polyaniline (PANI)

PANI remains a benchmark CP due to its reversible protonation chemistry and well-defined redox states (leucoemeraldine, emeraldine, pernigraniline) [45]. The emeraldine salt (ES) form exhibits the highest conductivity (~1–100 S cm^−1^) under acidic conditions, where protonic doping creates a delocalized polaron network. This proton-coupled electron transport renders PANI highly sensitive to pH, humidity, and analytes with proton-exchange capability, making it attractive for gas sensors and amperometric biosensors. Its ease of electropolymerization on various electrodes enables nanostructured coatings with excellent adhesion and fast electron transfer [46]. However, PANI’s limited stability in neutral or basic media and mechanical brittleness restrict its long-term operation in physiological environments. To mitigate this, researchers have developed PANI composites with biopolymers (chitosan, gelatin) or carbon nanomaterials that preserve protonic pathways while improving flexibility and biocompatibility [47,48]. Recent experimental demonstrations further confirm the pH-dependent electrochemical behavior of PANI in realistic biosensing scenarios. Wearable PANI-based hydrogel sensors and label-free aptasensors have achieved nanomolar detection limits for clinically relevant biomarkers, while simultaneously highlighting stability constraints and redox fatigue under prolonged operation [49,50].

### 3.3. Polypyrrole (PPy)

PPy is one of the earliest and most studied CPs in biosensing owing to its easy electropolymerization, moderate conductivity (~10 S cm^−1^), and robust film formation [51]. Electropolymerization allows precise thickness control and in situ encapsulation of enzymes or nanoparticles within the PPy matrix. The resulting films display good chemical stability and low background current, advantageous for amperometric sensors. PPy is also intrinsically porous and hydrophilic, enabling ion transport and analyte diffusion. This intrinsic porosity and hydrophilicity can be advantageous for sensing by facilitating ion transport and analyte diffusion, thereby enhancing sensitivity and response time. However, excessive porosity may also promote swelling, dopant leaching, and biofouling, negatively impacting signal stability and long-term reproducibility. These structural and electrochemical characteristics of PPy have been extensively validated in experimental sensing platforms. Impedimetric and amperometric PPy-based sensors have demonstrated nanomolar detection limits for biomolecules and metal ions, while also revealing practical limitations related to overoxidation, mechanical brittleness, and film delamination during repeated electrochemical cycling [52,53,54].

Its mechanical stiffness is higher than PEDOT:PSS but can be softened by co-polymerization with flexible monomers or dopants such as dodecyl sulfate [55]. PPy-based biosensors have demonstrated efficient detection of glucose, dopamine, and hydrogen peroxide, often through enzyme entrapment or nanocomposite integration [51,56].

Despite these advantages, a key limitation of PPy remains its tendency to undergo conductivity degradation under repeated redox cycling, often accompanied by partial delamination from metallic substrates. These effects are commonly attributed to volumetric changes during doping/dedoping processes and to relatively weak interfacial adhesion at the polymer–electrode interface. In recent years, hybridization with two-dimensional nanomaterials—most notably graphene derivatives and MXene nanosheets—has emerged as an effective strategy to mitigate these issues [57].

In PPy/graphene hybrids, strong π–π interactions between the conjugated PPy backbone and the sp^2^ carbon lattice of graphene or reduced graphene oxide provide mechanical reinforcement and improve adhesion to the underlying electrode. The graphene framework acts as a conductive scaffold that suppresses crack propagation and limits film delamination during electrochemical cycling. Experimental studies have shown that PPy/graphene nanocomposites exhibit enhanced electrical conductivity, lower charge-transfer resistance, and improved cycling stability compared to pristine PPy films, leading to higher sensitivity and improved signal stability in electrochemical sensing applications [58,59,60]. Similarly, PPy/MXene hybrids exploit the layered morphology and surface terminations (–O, –OH, –F) of MXenes (e.g., Ti_3_C_2_T_x_) to establish strong interfacial bonding with PPy chains. MXene nanosheets act both as mechanical anchors and as highly conductive pathways, alleviating volumetric stress during redox cycling and reducing the likelihood of delamination. Recent experimental work has demonstrated that PPy–MXene composite films display reduced signal drift, lower interfacial resistance, and improved reproducibility under repeated electrochemical operation. In biosensing contexts, such hybrids have enabled stable and sensitive detection by maintaining intimate electrical contact and structural integrity over prolonged use [61,62,63].

Overall, the hybridization of PPy with graphene or MXene nanosheets represents a powerful materials-engineering approach to overcome intrinsic limitations related to redox stability and mechanical integrity. By reinforcing adhesion, stabilizing conductivity under cycling, and improving charge-transfer kinetics, these nanocomposites significantly extend the operational lifetime and reliability of PPy-based biosensors.

### 3.4. Polythiophene (PTh) and Derivatives

Beyond PEDOT, other polythiophenes (PThs) provide a versatile materials platform for tuning the balance between backbone conjugation, side-chain polarity, and film morphology. The archetype poly(3-hexylthiophene) (P3HT) exhibits high crystallinity and charge-carrier mobility but inherently limited ionic permeability; accordingly, functionalized derivatives incorporating carboxylated or oligo-ethylene-glycol side chains have been developed to introduce hydrophilicity and accessible sites for biofunctionalization [64].

Electrochemical studies on P3HT films have experimentally demonstrated that controlled doping can modulate charge-carrier density and electrical conductivity over several orders of magnitude, thereby providing a clear materials-level basis for the use of polythiophene derivatives as active transduction layers in electrochemical sensing interfaces [65,66]. These properties, combined with their solution processability, have motivated their adoption in bioelectronic devices where electronic transport must coexist with ion-mediated processes.

Polythiophenes are particularly attractive for organic electrochemical transistors (OECTs) employed in bioelectronics, where mixed ionic–electronic conduction within the bulk channel governs transduction efficiency and signal amplification. Their compatibility with solution processing and printing further enables scalable fabrication of large-area sensor arrays and flexible platforms [67].

Importantly, beyond transistor-based architectures, polythiophene derivatives have also been directly implemented as active sensing films in electrochemical sensors. Experimental demonstrations include electropolymerized polythiophene interfaces for nitrite detection as well as poly(thiophene)-based composite electrodes supporting enzymatic glucose biosensing, with reported analytical figures of merit such as sensitivity, linear dynamic range, and limit of detection that are relevant for practical sensing applications [68,69].

In addition, engineered P3HT-based microelectrode interfaces have been experimentally validated for electrochemical monitoring in biologically relevant environments, including in vitro and in vivo platforms for small-molecule detection. These studies support the translational relevance of polythiophene-derived materials beyond proof-of-concept demonstrations and highlight their potential for integration into bioelectronic sensing systems operating under realistic conditions [70].

Although polythiophenes remain less extensively explored than PEDOT:PSS in electrochemical (bio)sensing, recent material developments point toward an expanding design space. New copolymers combining thiophene backbones with imidazolium units or sulfonated side chains are emerging as mixed ionic–electronic conductors with inherent ionic functionality and built-in biointeraction capabilities [71].

### 3.5. Emerging CP Composites and Hybrids

The last five years have witnessed an explosion of CP-based nanocomposites, integrating the electronic conductivity of CPs with catalytic, mechanical, or sorptive functionalities of nanofillers.

Carbon nanomaterials (graphene, CNTs, carbon black) enhance conductivity and surface area while providing mechanical reinforcement. For instance, a PEDOT/CNT–graphene oxide nanocomposite electrode has been experimentally demonstrated to significantly enhance charge transfer and electrocatalytic responses for simultaneous electrochemical detection of hydroquinone, catechol, and nitrite in real water samples, exhibiting low-nanomolar detection limits and good stability under practical conditions [72]. In addition, electrodes modified with electrochemically reduced graphene oxide and multi-walled carbon nanotubes have demonstrated sensitive simultaneous detection of catechol and hydroquinone, further illustrating the benefits of carbon nanostructures in composite sensing interfaces [73].

Similarly, electrochemical sensors incorporating gold nanoparticles and hydroxylated multiwalled carbon nanotubes/graphene composites have been shown to provide enhanced electrocatalytic activity and linear current response for the simultaneous detection of hydrazine and nitrite in aqueous samples [74]. Hybrid composites of conductive polymers and metal–organic frameworks have shown robust chemiresistive responses with enhanced stability and dynamic performance, reflecting the potential of MOF integration in CP sensing platforms [75].

Synergistic coupling between these components yields multi-modal transduction and improved sensitivity, though often at the cost of reproducibility and sustainability. The field is now shifting toward biopolymer–CP hybrids (e.g., chitosan-PEDOT:PSS, cellulose–PPy) that retain conductivity while introducing biodegradability and self-healing properties [76,77].

## 4. Electrochemical Sensing and Biosensing Mechanisms

**Figure 5:**
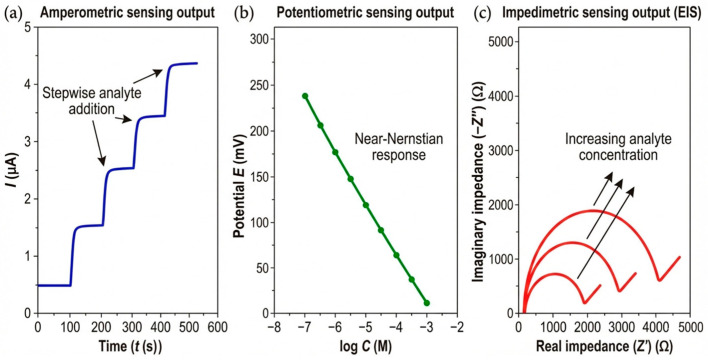
Typical experimental electrochemical outputs for conducting polymer-based sensing platforms. (**a**) Representative amperometric current–time response showing stepwise signal variations upon successive analyte additions. (**b**) Typical potentiometric calibration plot displaying a near-Nernstian dependence of electrode potential on the logarithm of analyte concentration. (**c**) Representative electrochemical impedance spectroscopy (EIS) Nyquist plots illustrating an increase in interfacial impedance after analyte binding. The panels illustrate experimentally observed signal trends reported across independent studies and do not represent data from a single device.

CPs function as the active transducing interface between a biochemical reaction and an electrical signal. Their ability to conduct both ions and electrons enables them to couple directly with redox processes occurring in solution, on immobilized enzymes, or at nanocomposite interfaces. Depending on the transduction mode—amperometric, potentiometric, voltammetric, or impedimetric—the CP participates in charge transfer either as a redox mediator, a charge-storage matrix, or a dynamic ionic conductor. To illustrate how these transduction mechanisms translate into experimentally measurable signals, Figure 5 reports representative electrochemical outputs typically observed for CP-based sensors operating in amperometric, potentiometric, and impedimetric modes.

**Figure 6:**
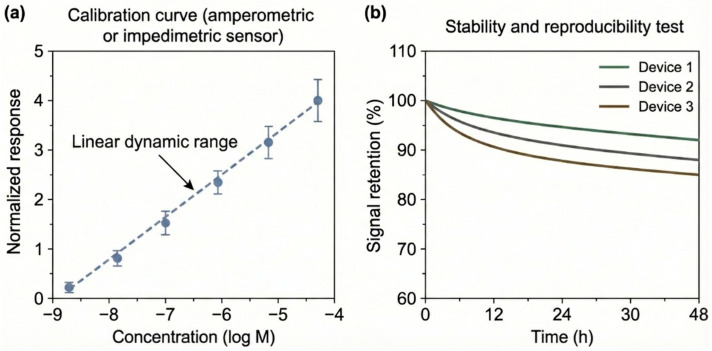
Representative experimental performance metrics for conducting polymer-based electrochemical sensors. The figure summarizes typical calibration behavior, linear dynamic range, and signal stability commonly reported for CP-based electrochemical sensors operating in amperometric, potentiometric, and impedimetric modes. The trends shown are derived from primary experimental literature and illustrate how sensing performance is quantitatively evaluated across different transduction mechanisms. The figure is intended to highlight representative performance metrics rather than absolute record values, which are instead discussed within the benchmarking framework provided in Table 2.

Beyond the qualitative illustration of signal outputs, Figure 6 summarizes representative experimental performance metrics—such as calibration behavior, linear dynamic range, and signal stability—commonly used to evaluate CP-based electrochemical sensors across different transduction modes. Together, Figure 5 and Figure 6 anchor the subsequent discussion to experimentally observed signal outputs and quantitative performance metrics reported in state-of-the-art CP-based electrochemical sensors. The data trends depicted in these figures are extracted from and consistent with primary experimental studies, and are intended to reflect real measurement behavior rather than conceptual abstractions.

**Table 2:** Benchmarking of CP-Based Electrochemical (Bio)Sensors: Performance Envelopes and Trade-Offs.

Polymer System	Dominant Sensing Modalities	Typical LOD (Order of Magnitude)	Linear Range (Typical)	Operational Stability *	Reproducibility (Typical RSD)	Dominant Failure Modes	Representative References (This Review)
PEDOT:PSS	Amperometric, EIS, OECT	nM–µM (enzymatic); fM–pM (EIS)	2–4 decades	Hours–days (continuous operation); weeks (intermittent use)	5–10% (intra-batch)	Dopant migration, hydration-induced drift, biofouling	[37,38,39,40,41,42,48,49,50,73]
Polypyrrole (PPy)	Amperometric, voltammetric, EIS	nM–µM	1–3 decades	Hours–days	10–20%	Overoxidation, delamination, conductivity loss	[47,50,51,63,78]
Polyaniline (PANI)	Amperometric, potentiometric	nM–µM (acidic media)	1–3 decades	Hours–days (pH-dependent)	10–25%	pH instability, redox fatigue	[43,44,45,46,64]
Polythiophenes/OMIECs	OECT, EIS	pM–nM	2–4 decades	Days–weeks	5–15%	Synthetic and processing complexity, batch-to-batch variability, processing variability	[30,34,73]

* Operational stability refers to signal retention under continuous or repeated operation in aqueous or biological media, rather than shelf life. The reported values represent representative performance envelopes rather than record metrics, as they are extracted from independent experimental studies employing different device architectures, electrolytes, and testing protocols. Accordingly, the table is intended to highlight realistic trade-offs among sensitivity, linear range, and operational stability across polymer families and sensing modalities, rather than to provide absolute rankings.

### 4.1. Amperometric Sensors

Amperometric devices measure the current generated by oxidation or reduction in an analyte at a fixed potential. In CP-based electrodes, the polymer acts as a mediator layer that enhances electron transfer between the active site of an enzyme or catalytic nanoparticle and the underlying electrode [9]. For example, recent experimental implementations further demonstrate that PEDOT:PSS-based amperometric glucose biosensors achieve low detection limits and stable operation in complex biological matrices, including saliva and interstitial fluids, when integrated with nanostructured catalysts or redox-active nanoparticles [56,79,80]. The polymer’s high surface area and redox capacity accelerate this step, improving sensitivity and reducing the overpotential for H_2_O_2_ oxidation. Similarly, PANI and PPy films doped with redox mediators (e.g., ferricyanide, ferrocene) allow direct wiring of redox enzymes, bypassing diffusional mediators [51,81]. The ion-permeable structure of CPs permits rapid diffusion of reactants and by-products, while their electronic network ensures fast electron shuttling [82]. Amperometric CP-based biosensors therefore combine enzymatic selectivity with electrocatalytic amplification, achieving detection limits in the nM–µM range for glucose, lactate, dopamine, and various metabolites [51,79,81,83]. However, because the current is directly proportional to analyte flux, the stability of the CP redox state and the prevention of fouling are crucial for reproducibility over repeated cycles.

### 4.2. Potentiometric Sensors

Potentiometric CP-based sensors rely on the measurement of open-circuit potential arising from ion-exchange or redox equilibrium at the CP–electrolyte interface. In this configuration, the CP film behaves as a mixed conductor membrane whose potential responds to the activity of specific ions or charged analytes [46]. Protonated PANI and doped PPy films exhibit well-defined potential–pH dependences, making them useful for solid-state pH electrodes or ion-selective membranes [84]. The slope of potential versus log[H^+^] often approaches the Nernstian limit (≈59 mV per decade at 25 °C), evidencing reversible proton-coupled electron transfer within the polymer matrix. Such near-Nernstian responses have been experimentally validated in solid-state PANI-based potentiometric electrodes, including flexible carbon-cloth and composite film platforms, confirming their suitability for real-world pH and ion sensing [46,84]. In biosensing, potentiometric CP electrodes can be functionalized with ionophores, enzymes, or aptamers whose activity modulates local ion concentration (e.g., NH_4_^+^, H^+^, Cl^−^) [85,86]. The resulting potential shift provides a label-free measure of analyte concentration. PEDOT:PSS films with immobilized urease, for instance, exhibit potential changes proportional to urea concentration due to pH variation upon enzymatic hydrolysis [87]. Potentiometric CP sensors are particularly suitable for low-power or passive readout systems, though they require stringent control of reference electrodes and ionic strength to avoid drift.

### 4.3. Voltammetric and Differential Pulse Techniques

Voltammetric methods—cyclic voltammetry (CV), differential pulse voltammetry (DPV), or square-wave voltammetry (SWV)—exploit the redox activity of CPs to probe specific analytes. In such systems, CPs can function both as electron-transfer mediators and as substrates for adsorption or catalysis. For example, dopamine oxidation on PEDOT:PSS or PPy films produces a distinct anodic peak that is well separated from interferents such as ascorbic acid or uric acid, due to the polymer’s charge-selective and π–π interaction sites. Experimental voltammetric studies using PPy-based nanocomposites have demonstrated clear peak separation and selective dopamine detection in the presence of common electroactive interferents, validating the charge-selective and π–π interaction mechanisms attributed to CP films [51,88]. For example, a PEDOT-PPy hybrid composite electrode exhibited well-defined CV and DPV peaks for dopamine oxidation with a low detection limit (5 nM) and clear discrimination from common interferents in phosphate buffer, validating the enhanced electrocatalytic and charge-transfer characteristics of CP composites [78]. Incorporation of carbon nanomaterials further sharpens these redox features by improving conductivity and electron density at the interface [88]. Similarly, PEDOT/graphene oxide composite electrodes synthesized in deep eutectic solvents have been shown to enable simultaneous voltammetric detection of acetaminophen and ascorbic acid with well-resolved peaks and suitable analytical figures of merit [89]. Voltammetric CP sensors thus excel in selectivity and multi-analyte detection, enabling fingerprint recognition of electroactive species in biological fluids. Their performance depends on the film’s electrochemical window, capacitance, and density of redox sites, all of which are tunable through doping and nanostructuring [83].

### 4.4. Impedimetric (Capacitive) Sensors

Electrochemical impedance spectroscopy (EIS) provides a powerful, label-free approach to detect surface binding or conformational changes in bioreceptors immobilized on CP films. In these systems, the CP behaves as a porous ionic conductor whose charge-transfer resistance (R_ct_) and double-layer capacitance (C_dl_) vary with analyte binding. For example, when antibodies immobilized on a PEDOT:PSS/Au nanocomposite capture antigen molecules, the insulating biomolecular layer increases R_ct_, leading to a quantifiable impedance change.

Because EIS probes both resistive and capacitive components, it is highly sensitive to surface coverage, fouling, and hydration. CPs enhance these sensors by providing flexible, hydrated matrices that accommodate biofunctional layers without delamination. The presence of multiple time constants in the impedance spectra reflects distinct physical processes. The high-frequency response is typically associated with the polymer–electrode interface and film resistance/capacitance, whereas the low-frequency arc corresponds to charge-transfer resistance and mass transport. Notably, some CP-based EIS sensors intentionally exploit variations in capacitive behavior arising from surface binding or hydration changes, rather than relying exclusively on R_ct_ modulation. Typical detection limits reach at femtomolar (fM) and even attomolar (aM) levels for protein biomarkers (e.g., PSA, CRP, HER2) [90,91,92]. However, impedance spectra can be influenced by film inhomogeneity or ionic drift, necessitating appropriate equivalent-circuit modeling and environmental stabilization.

### 4.5. Signal Transduction Pathways and Hybrid Mechanisms

While the categories above provide a convenient classification, many modern CP-based sensors operate through hybrid or multimodal mechanisms. For instance, OECT-type biosensors use a CP channel (often PEDOT:PSS or P3CPT) whose conductivity changes with ionic gating from analyte interactions, effectively merging amperometric and capacitive principles [93]. These hybrid transduction pathways have been experimentally validated in OECT-based biosensors for clinically relevant biomarkers, where ionic gating and electronic transport are quantitatively correlated under realistic operating conditions [87,93]. Similarly, CP hydrogels in wearable devices may simultaneously record electrochemical and mechanical (pressure/strain) signals, reflecting the same ionic pathways governing conductivity [80,94,95,96].

**Figure 7:**
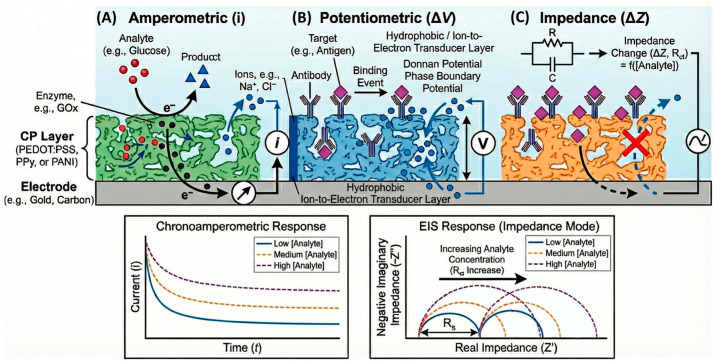
Schematic representation of conducting-polymer-based electrochemical biosensors. The upper panel illustrates the sensing interface and the three main transduction modes: (**A**) amperometric sensing, (**B**) potentiometric sensing, and (**C**) impedimetric sensing. This schematic is intended to provide a conceptual framework for the sensing interfaces and transduction mechanisms discussed in Section 4 and does not represent experimental data. Representative experimental outputs and quantitative performance metrics corresponding to these transduction modes are reported in Figure 5 and Figure 6.

The emergence of self-powered systems coupling triboelectric or piezoelectric effects with CP electrodes further expands the transduction landscape, enabling energy-autonomous sensing in flexible and transient platforms [97]. These multimodal architectures underline the importance of correlating charge transport, ion dynamics, and interfacial chemistry—the triad that ultimately dictates sensor performance and reliability. For clarity, the main sensing interfaces and electrochemical transduction pathways discussed in Section 4.1, Section 4.2, Section 4.3 and Section 4.4 are schematically summarized in Figure 7, which provides a conceptual overview complementary to the representative experimental outputs shown in Figure 5 and Figure 6.

Throughout this review, conceptual figures are intentionally used to synthesize and contextualize experimental trends reported across a broad primary literature, while representative signal outputs, performance metrics, and benchmarking data are explicitly anchored to experimental studies (Figure 5 and Figure 6, and Table 2).

Although Section 4.1, Section 4.2, Section 4.3 and Section 4.4 describe distinct electrochemical transduction mechanisms, their performance cannot be meaningfully compared based on sensitivity alone. Each modality exhibits characteristic trade-offs between limit of detection, linear dynamic range, temporal stability, and susceptibility to drift or fouling. For this reason, quantitative benchmarking across both polymer families and sensing modalities is required to assess relative advantages under realistic operating conditions, as discussed in Section 6 and Section 9 and summarized in Table 2.

For clarity, the main sensing interfaces and electrochemical transduction pathways discussed in Section 4.1, Section 4.2, Section 4.3 and Section 4.4 are schematically summarized in Figure 7, which is intended as a conceptual aid and not as a representation of experimental data.

## 5. Engineering Conducting Polymers for Enhanced Performance

The fundamental mixed ionic–electronic nature of CPs provides a unique foundation for electrochemical sensing, yet their raw forms rarely exhibit optimal stability, selectivity, or reproducibility. To achieve high-performance devices, extensive materials engineering is required across multiple hierarchical levels—ranging from molecular doping and nanostructuring to surface functionalization and antifouling.

**Figure 8:**
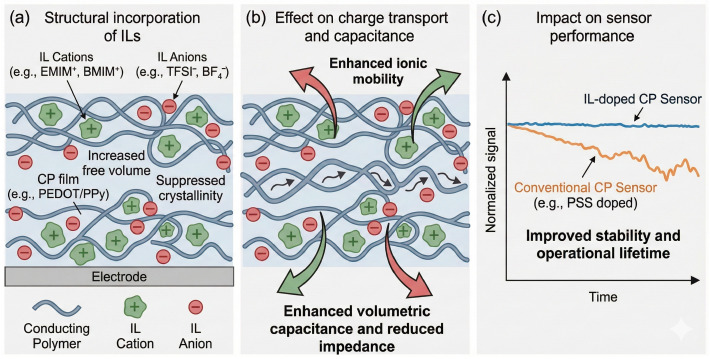
Influence of ionic liquids on (**a**) structure, (**b**) transport and (**c**) electrochemical performance of conducting polymer sensors. The figure is constructed from trends reported in primary experimental literature and is intended to summarize experimentally observed effects rather than depict a single device or conceptual model.

**Figure 9:**
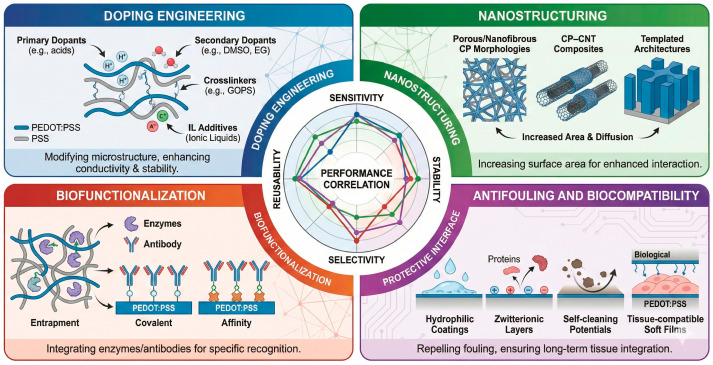
Engineering-oriented synthesis of performance correlations in conducting polymer-based electrochemical sensors. The figure integrates experimentally established trends reported in the literature to illustrate how doping engineering, nanostructuring, biofunctionalization, and antifouling strategies collectively influence key performance descriptors, including sensitivity, selectivity, stability, and reusability. The radar plot represents qualitative correlations rather than absolute metrics and is intended to provide a unifying interpretative framework derived from primary experimental studies, rather than depict a single device or dataset.

This section highlights four major design strategies that have proven most effective in optimizing the sensing performance of CP-based electrodes. Figure 8 shows conceptually what is the influence of ionic liquids on structure, transport and electrochemical performance of conducting polymer sensors. To address limitations of purely conceptual illustrations this section is supported by Figure 9, which summarizes experimentally established engineering strategies and their quantitative impact on sensing performance. Figure 9 consolidates trends and design effects reported across primary experimental studies, providing a data-grounded framework for interpreting how doping, nanostructuring, biofunctionalization, and antifouling strategies influence sensitivity, stability, and reproducibility.

### 5.1. Doping and Secondary Dopants

In PEDOT:PSS systems, the sulfonated polystyrene (PSS^−^) primary dopant plays a dual role as charge-compensating counter-ion and microstructural templating agent. Recent experimental investigations have shown that exposure to aqueous or bio-relevant electrolytes can induce dopant redistribution, water uptake, and interfacial aging, leading to progressive changes in impedance and electrical drift over time. These effects are directly linked to the mobility of PSS^−^-associated ionic species and to local microstructural inhomogeneities within the conducting network [98]. In polypyrrole-based films, electrical conductivity is governed by the incorporation of inorganic or surfactant-derived anions during oxidative polymerization. Experimental studies have demonstrated that the nature of the dopant critically affects hygroscopicity, ion retention, and long-term electrical stability, with soft or mobile dopants promoting ionic leaching and conductivity drift under humid or electrochemical conditions. Such behavior highlights the intrinsic trade-off between high initial conductivity and operational stability in PPy systems [55]. In polyaniline, charge transport is enabled through protonic doping of the emeraldine base, typically achieved using strong acids. Recent experimental reports indicate that proton migration, dopant loss, and environmental sensitivity can lead to reversible or irreversible dedoping phenomena, particularly under cyclic electrochemical or aqueous exposure. These processes result in temporal variations in conductivity and limit the long-term reliability of PANI-based sensing interfaces [46].

To overcome these drawbacks, researchers employ secondary dopants—polar organic solvents or ionic additives that modify the morphology and enhance conductivity by up to three orders of magnitude. EG, DMSO, sorbitol, and ILs induce phase segregation between PEDOT-rich and PSS-rich domains, improving π–π stacking and connectivity [12,99]. ILs increase the effective dielectric constant and ionic polarizability of the CP matrix, enhancing volumetric capacitance and stabilizing ionic transport. This results in reduced impedance, improved charge injection, and enhanced sensitivity, particularly in low-voltage and impedance-based electrochemical sensing configurations. Moreover, ILs such as 1-ethyl-3-methylimidazolium bis(trifluoromethanesulfonyl)imide (EMIM TFSI) and 1-butyl-3-methylimidazolium tetrafluoroborate (BMIM BF_4_) simultaneously act as plasticizers and ionic reservoirs, maintaining hydration and ionic mobility under dry conditions [100]. These effects are summarized comparatively in Figure 8, which highlights how different doping and secondary-doping strategies quantitatively affect conductivity, volumetric capacitance, and operational stability in CP-based sensors.

Covalent crosslinkers (e.g., glycidoxypropyltrimethoxysilane, GOPS) stabilize PEDOT:PSS films against delamination and dopant loss, essential for long-term operation in aqueous or biological media [101,102]. Fine-tuning the dopant type, concentration, and distribution allows control over conductivity, work function, and surface energy—parameters that directly influence electron-transfer kinetics, analyte adsorption, and biocompatibility.

### 5.2. Nanostructuring and Surface Engineering

Morphology critically dictates the electrochemical activity of CP electrodes. Nanostructuring enhances performance by increasing the effective surface area, ion diffusion rate, and density of electroactive sites [103].

Common strategies include:

- Template-assisted polymerization enables the formation of nanostructured PPy architectures with increased surface roughness and accessible electroactive domains, which promote faster ion transport and enhanced electrochemical activity compared to dense films [55].
- Self-assembled PEDOT:PSS microgel and hydrogel networks generate interconnected porous pathways that facilitate ion diffusion throughout the bulk of the electrode, effectively increasing the density of electroactive sites and the volumetric capacitance [29].
- Nanocomposites combining CPs with carbon nanomaterials (graphene, CNTs, carbon black) or 2D materials (MXenes) to create percolative electronic pathways [104,105,106].
- Nanofibrous architectures obtained by electrospinning or printing provide highly accessible conductive pathways and mechanically compliant meshes, leading to enhanced electrochemical performance through increased interfacial area and improved ion diffusion kinetics [18].

Nanostructured CPs exhibit faster electron transfer rates (k_0_) and higher (C_dl_), translating into improved signal-to-noise ratios and lower detection limits in amperometric and impedimetric modes. However, excessive porosity or uncontrolled swelling can compromise mechanical integrity and reproducibility. Thus, hierarchical order—mesoporous but structurally cohesive architectures—is emerging as the optimal compromise for biosensing.

### 5.3. Biofunctionalization and Bioreceptor Immobilization

One of the most powerful aspects of CPs is their chemical adaptability, which enables integration of biological recognition elements directly within or atop the conductive matrix.

Biofunctionalization strategies fall into three major categories:

1. Physical entrapment—Enzymes, antibodies, or DNA strands are immobilized during electropolymerization (e.g., PPy–glucose oxidase), ensuring intimate electrical contact while retaining activity [83].
2. Covalent coupling—Surface functional groups (–COOH, –NH_2_, –OH) introduced via copolymerization or plasma treatment enable stable bonding of biomolecules through EDC/NHS or glutaraldehyde chemistry [107].
3. Affinity interactions—Biorecognition elements can be anchored via streptavidin–biotin, peptide tags, or electrostatic adsorption on PSS-rich PEDOT:PSS films [108].

In each case, the goal is to preserve the native bioactivity while optimizing electron and ion transfer at the bio–polymer interface. Hybrid CPs with metal nanoparticles (Au, Pt, Ag) or redox mediators (ferrocene, quinones) further facilitate catalytic turnover and reduce overpotentials in enzymatic sensors [56]. Recent work has demonstrated biofunctional CP hydrogels, which maintain hydration and ionic conductivity while supporting cell adhesion and enzymatic reactivity—ideal for wearable or implantable biosensors [11,81]. In such systems, control over the microenvironmental pH, ionic strength, and diffusion length becomes essential to balance sensitivity with long-term stability.

### 5.4. Antifouling and Biocompatible Interfaces

For real biological matrices such as blood, serum, or sweat, biofouling remains one of the most severe challenges. Adsorption of proteins and cells onto the CP surface leads to signal drift and loss of sensitivity [23].

Several engineering strategies have been developed to mitigate this effect:

- Surface passivation with hydrophilic or zwitterionic polymers (polyvinyl alcohol, MPC, sulfobetaines) to form hydration layers that repel nonspecific adsorption [109].
- Incorporation of biopolymers (chitosan, alginate, gelatin) within CP matrices, combining natural antifouling with improved softness and biodegradability [107].
- Dynamic coatings and self-cleaning interfaces, where potential pulses or electrochemical redox cycling remove adsorbed foulants [110].
- Nano- and micro-patterned surfaces, exploiting topographical cues to minimize effective contact area with fouling species [111].

In parallel, ensuring cytocompatibility and tissue integration has become central for wearable and implantable biosensors. PEDOT:PSS, for example, is now routinely used as a neural interface coating, displaying low impedance and reduced inflammatory response over months of implantation [112]. The emerging paradigm shifts from inert surfaces to “bio-intelligent” interfaces, where CPs not only resist fouling but actively sense or modulate their biochemical surroundings.

### 5.5. Balancing Performance and Stability

While these engineering approaches markedly improve sensitivity and selectivity, they often introduce trade-offs:

- Strong dopants may increase conductivity but accelerate degradation.
- High surface area enhances sensitivity but exacerbates fouling.
- Crosslinking improves stability but reduces flexibility.

Addressing these competing effects requires multiscale optimization—linking molecular design, processing conditions, and operating environment to predictable sensor performance.

Based on the experimentally validated engineering strategies discussed in this section, Figure 9 provides a consolidated synthesis of how doping, nanostructuring, biofunctionalization, and antifouling approaches collectively influence sensitivity, selectivity, stability, and reusability in CP-based electrochemical sensors.

## 6. CP-Based Electrochemical Biosensors by Target Class

To facilitate comparison across different polymer systems and sensing modalities, the following sections report representative quantitative performance metrics—such as limit of detection, linear range, and operational stability—highlighting how these values cluster into characteristic performance envelopes rather than isolated record values. This approach enables identification of material- and architecture-dependent trade-offs that are not readily apparent from single-device demonstrations.

The translation of CP science into functional electrochemical biosensors has produced a broad spectrum of devices addressing chemical, biochemical, and environmental targets. Despite the diversity of analytes, all CP-based systems rely on a few common features: (i) efficient electron/ion coupling between the analyte or recognition element and the polymer matrix; (ii) stability of the transducing layer under realistic operating conditions; and (iii) compatibility with biofunctional coatings or composites that extend selectivity. This section organizes representative progress by target class, providing quantitative comparisons and identifying recurring design principles.

**Figure 10:**
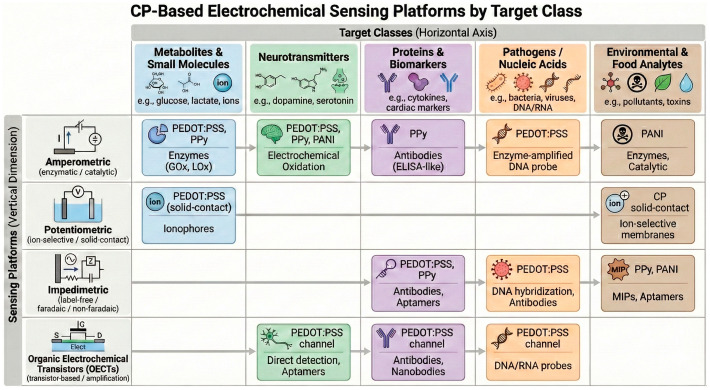
Overview of conducting polymer-based electrochemical sensing platforms organized by analyte target class and transduction mode. The figure summarizes typical associations between target categories (metabolites and small molecules, neurotransmitters, proteins and biomarkers, pathogens/nucleic acids, and environmental and food analytes) and the most commonly reported sensing platforms (amperometric, potentiometric, impedimetric, and organic electrochemical transistors), together with representative polymer systems and biorecognition strategies. The schematic is intended as a conceptual guide to the sensing platforms discussed in Section 6 and does not represent individual devices, datasets, or quantitative performance metrics. Abbreviations: GOx, glucose oxidase; LOx, lactate oxidase; MIPs, molecularly imprinted polymers.

For clarity, Figure 10 provides an overview of the main CP-based electrochemical sensing platforms and their typical association with different target classes, serving as an orientation map for the sensing strategies discussed in Section 6.1, Section 6.2, Section 6.3, Section 6.4, Section 6.5 and Section 6.6.

### 6.1. Metabolites and Small Molecules

Metabolite sensing remains the most mature field for CP-based electrochemical biosensors, particularly for glucose, lactate, uric acid, and hydrogen peroxide. These analytes serve as benchmark systems for evaluating charge transfer and enzymatic immobilization in CP matrices.

- Glucose sensors: PEDOT:PSS and PPy are the most widely used CPs for glucose oxidase (GOx) immobilization. The polymer provides a hydrophilic matrix allowing diffusion of both glucose and O_2_, while the redox-active backbone mediates electron transfer between the enzyme and electrode [25]. Incorporation of Au or Pt nanoparticles into PEDOT:PSS films further lowers the overpotential for H_2_O_2_ oxidation and extends linearity down to µM levels.
- Lactate and uric acid: PANI and PPy have demonstrated superior catalytic activity toward lactate oxidase and uricase reactions, respectively, especially when doped with protonic acids or ILs that enhance ionic exchange [113,114].
- Hydrogen peroxide and ROS detection: CP composites with reduced graphene oxide (rGO) or metal nanostructures (AuNW/rGO–PEDOT:PSS) can directly sense reactive oxygen species released from living cells with LODs < 10 nM, offering real-time monitoring of oxidative stress [115].

PEDOT:PSS’s soft, hydrated morphology ensures biocompatibility, making these systems ideal for integration into wearable patches and microfluidic sweat analyzers.

### 6.2. Neurotransmitters and Neurochemicals

The detection of dopamine (DA), serotonin (5-HT), norepinephrine, and ascorbic acid exemplifies the synergy between CPs’ electroactivity and molecular selectivity. Dopamine oxidation occurs at potentials overlapping with other redox species; thus, charge-selective CP films (PEDOT:PSS, PPy) are crucial to discriminate cationic DA from anionic interferents (ascorbate, urate). Functionalization with negatively charged dopants (e.g., PSS^−^, Nafion) or π–π-interacting groups enhances selectivity and signal resolution [116].

In PEDOT:PSS/graphene nanocomposites, synergistic effects between the polymer’s ionic conduction and graphene’s high surface area yield detection limits down to 8 nM for dopamine [117]. Flexible CP electrodes printed on PET or PDMS substrates enable on-skin or in vivo monitoring of neurotransmitter release, combining electrochemical and mechanical compliance [112]. Moreover, polymer coatings on microelectrode arrays reduce impedance and noise, improving signal fidelity in neurophysiological recordings [118].

### 6.3. Protein Biomarkers and Disease Diagnostics

CP-based biosensors have rapidly expanded into clinical diagnostics, detecting biomarkers such as prostate-specific antigen (PSA), cardiac troponin (cTnI), C-reactive protein (CRP), HER2, and glucose transporter proteins. PEDOT:PSS and PPy are favored matrices for antibody or aptamer immobilization, offering abundant functional sites and ionic permeability [9]. For instance, PEDOT:PSS/Au-based nanocomposite HER2 electrochemical biosensors have achieved pg mL^−1^ detection limits and stable operation in serum over several weeks [27].

PANI/AuNP immunosensors for CRP demonstrated reproducible response across multiple cycles with minimal signal drift. Impedimetric detection dominates in this category, where the binding of large biomolecules modulates the R_ct_ [107,119]. Hybrid CPs incorporating biopolymer hydrogels maintain hydration and reduce fouling, enabling detection in undiluted serum or saliva [95]. Such designs bridge bioelectronic interfaces and point-of-care diagnostics, paving the way toward miniaturized, multiplexed biosensor arrays.

### 6.4. Pathogens, Nucleic Acids, and Molecular Recognition

CPs are increasingly employed in label-free DNA/RNA and pathogen detection, leveraging their sensitivity to surface charge alterations. In aptamer-functionalized PEDOT:PSS or PPy electrodes, target binding induces conformational or electrostatic changes measurable as shifts in impedance or potential [108,120]. Examples include PPy–AuNP composites for *E. coli* and SARS-CoV-2 RNA detection, achieving a LOD of 3.16 × 10^−17^ M (31.6 aM, attomolar) [121]. This result has been obtaining through a mechanism based on the conformational change in the aptamer upon binding to the nucleocapsid protein (N-protein) or viral RNA. This folding brings the DNA phosphate backbone charges closer or further away from the PPy surface, modulating the surface doping and thus the reported conductivity or impedance.

PEDOT:PSS-based DNA sensors exploit the differential binding of single- vs. double-stranded DNA to modulate charge injection efficiency. The soft, hydrated structure of CP hydrogels supports the maintenance of biomolecular activity and facilitates microfluidic integration for rapid molecular diagnostics [95].

### 6.5. Environmental and Food Analysis

Environmental sensing extends the scope of CPs beyond biomedicine, applying their redox activity and ion-exchange capacity to detect pollutants, heavy metals, pesticides, and food contaminants.

- Heavy metals: PANI and PPy modified with chelating ligands (EDTA, crown ethers) act as preconcentrators for Pb^2+^, Cd^2+^, or Hg^2+^, allowing detection via anodic stripping voltammetry [122].
- Pesticides: acetylcholinesterase (AChE)-based electrochemical biosensors exploiting enzyme inhibition have been widely implemented on PEDOT:PSS-modified electrodes for the selective detection of organophosphates in food and water matrices [123].
- Food freshness and spoilage: CP-based impedimetric sensors detect biogenic amines or bacterial metabolites, serving as electronic noses for smart packaging [124,125].

In environmental matrices, the challenge lies in maintaining stability and selectivity under complex ionic conditions. Advances in CP nanocomposites and antifouling coatings have significantly improved reproducibility, moving toward deployable field sensors.

### 6.6. Emerging Trends Across Analyte Classes

A cross-comparison of recent data reveals several unifying trends:

- PEDOT:PSS composites frequently offer a favorable balance of conductivity, mechanical flexibility, and biocompatibility relative to many alternative materials, and are widely adopted as transduction/immobilization matrices in diverse biosensors. However, performance is analyte- and architecture-dependent, and in some cases other polymers or composites may deliver superior trade-offs for specific targets or operating conditions.
- PPy and PANI are among the most commonly used CPs for enzymatic biosensors because of facile electropolymerization, good film-forming ability, and effective enzyme entrapment. Nonetheless, many reports note stability challenges in neutral/physiological media, motivating the use of composites, mediators, or alternative architectures to enhance robustness under near-neutral conditions.
- Nanocomposite and conductive hydrogel systems increasingly enable soft, flexible, and wearable/transient biosensors, supporting opportunities for continuous biochemical monitoring. Reported advantages include improved mechanical compliance, biocompatibility, and, in some cases, enhanced signal transduction; challenges remain for long-term stability, biocompatibility in vivo, and reproducible large-area fabrication.
- Impedance-based (label-free) detection has gained prominent traction in recent years and is frequently highlighted in reviews as a scalable, low-power readout for many CP- and hydrogel-based biosensors. However, amperometric and voltammetric approaches continue to be widely reported for enzymatic glucose sensors, including GOx-based systems, so impedance-based methods have not universally surpassed amperometry in the literature. The relative prevalence is method- and application-dependent and shows an increasing but not exclusive trend toward impedance.

## 7. CPs in Wearable, Implantable and Soft Bioelectronics

The recent evolution of bioelectronic technologies has profoundly transformed the landscape of electrochemical sensing. Devices are no longer confined to rigid electrodes immersed in controlled solutions but are now expected to operate on the skin, within tissues, or even to disappear after use. In this paradigm, CPs—notably PEDOT:PSS, PPy, and CP hydrogels—are at the forefront, offering a rare combination of electrical conductivity, ionic permeability, softness, and biocompatibility unmatched by metals or carbon-based conductors. Their capacity to transduce ionic signals into electronic currents under mechanical deformation enables a new generation of wearable, implantable, and transient biosensors, seamlessly merging electronics with biological matter.

**Figure 11:**
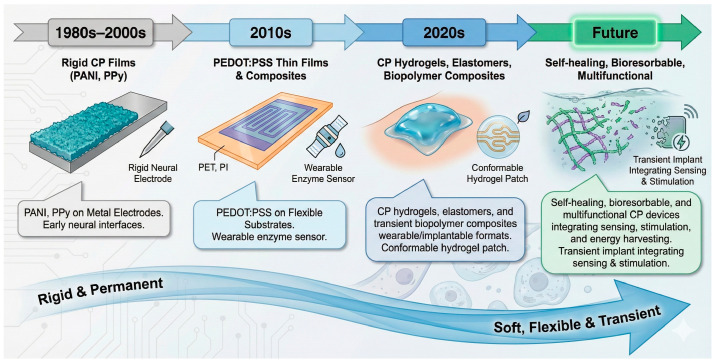
Evolution of conducting polymer-based electrochemical sensing platforms. The schematic summarizes the evolution of conducting polymer (CP) sensing technologies from early rigid PANI and PPy electrodes (1980s–2000s), through flexible PEDOT:PSS thin films and wearable sensors (2010s), to recent soft hydrogels, elastomers, and biopolymer composites (2020s). Future directions emphasize self-healing, bioresorbable, and multifunctional CP systems integrating sensing, stimulation, and energy harvesting, highlighting the transition from rigid, permanent devices to soft, flexible, and transient bioelectronic platforms.

To provide a unifying perspective on this transition, Figure 11 summarizes the historical evolution of conducting polymer-based bioelectronic platforms, highlighting the progressive shift from rigid and permanent CP films toward soft, flexible, and transient systems designed for wearable and implantable sensing.

### 7.1. Wearable Electrochemical Biosensors

Wearable CP sensors based on PEDOT:PSS and related CPs are increasingly deployed for continuous, non-invasive monitoring of biomarkers in body fluids such as sweat, saliva, interstitial fluid (ISF), and tears, enabling real-time health feedback in daily life and clinical settings [126]. The broad adoption of PEDOT:PSS in wearable platforms stems from its aqueous-processability and compatibility with flexible substrates, including PET, PI, and TPU, which facilitates scalable fabrication and comfortable, conformal wearables without compromising device performance

PEDOT:PSS hydrogels—obtained by partial replacement of PSS with crosslinkers (e.g., PEGDA, GOPS) or ILs—exhibit electronic conductivities on the order of 10 S cm^−1^ with water contents up to ~75%, enabling conformal skin adhesion and stable electrophysiological signal acquisition under motion [115]. Such hydrogels can host enzymes (lactate oxidase, glucose oxidase) or aptamers for metabolite detection in sweat, achieving sub-micromolar detection limits while maintaining stable operation over several hours of wear [80]. Flexible PEDOT:PSS/graphene-oxide composites show stable and repeatable sensing response under dynamic bending over ≥1000 cycles, indicating strong interlayer adhesion and negligible delamination [127]. PEDOT:PSS-based printed sensors have also demonstrated excellent mechanical durability, maintaining performance after 10,000 bending cycles at a 5 mm bending radius [42]. Flexible devices based on PEDOT:PSS/graphene or PEDOT:PSS/CNT composites exhibit high mechanical resilience, maintaining stable electrical and sensing performance under small bending radii (≈5 mm) and after thousands of bending cycles. These platforms can be seamlessly integrated with microfluidic sweat collectors or textile electrodes, forming soft electronic skins for metabolic and hydration monitoring [128]. Wearable electrochemical systems increasingly employ OECT-type configurations, where the CP channel acts as a volumetric transducer modulated by ionic fluxes in body fluids. Such devices enable low-voltage (<1 V), low-power operation, critical for mobile or self-powered biosensing.

### 7.2. Implantable and Neural Interfaces

Among all applications, implantable CP-based biointerfaces epitomize the synergy between electronic conductivity and biological softness. PEDOT:PSS coatings on metal microelectrodes (e.g., Au) markedly lower interfacial impedance (e.g., tens of kΩ at 1 kHz for PEDOT:PSS-coated Au microelectrodes) while improving charge storage/injection metrics, owing to the polymer’s high volumetric capacitance and conformal, mixed ionic–electronic charge storage that mitigates electrode–tissue mismatch and noise [112,129]. Long-term in vivo studies demonstrate stable operation of PEDOT:PSS electrodes for months, with minimal inflammatory response compared to bare metals [130].

In neural recording and stimulation, CPs provide bidirectional interfaces capable of sensing neuronal activity (µV range) and delivering electrical stimuli without Faradaic degradation [131]. Recent designs in CP bioelectronics employ PEDOT:PSS-based hydrogels that offer soft, tissue-like mechanical properties amenable to conformal neural interfacing, and elastomer-integrated PEDOT composites such as PEDOT:sSEBS demonstrate very high stretchability (>100% elongation at break), enabling conformal electrodes on stretchable substrates with strains well beyond 30% [132,133]. Conductive polypyrrole-chitosan (PPy-CHI) hydrogels have been developed as electrically conductive biomaterials that can support sustained release of therapeutic exosomes while maintaining electrical conductivity, suggesting their utility in bioelectronic scaffolds where conductive polymer/biopolymer composites enhance functional integration with tissue [134].

### 7.3. Transient and Biodegradable CP-Based Electronics

The push toward sustainable and biodegradable technologies has inspired a new frontier—transient CP-based biosensors that operate for a defined period and then dissolve harmlessly. Such devices are essential for temporary implants, environmental monitors, or single-use diagnostics. PEDOT:PSS films incorporated into cellulose, silk fibroin, or bacterial cellulose substrates retain electrical performance while being biodegradable under mild enzymatic conditions [135]. When combined with water-soluble dopants or ILs, they can be designed to self-disassemble after exposure to physiological fluids or water. Hybrid systems using chitosan–PEDOT:PSS or alginate–PPy demonstrate full disintegration within days to weeks, enabling transient recording or sensing without retrieval surgery [136]. This field aligns with the broader concept of green bioelectronics, where sustainable processing and end-of-life recovery are integral to device design.

### 7.4. Integration Challenges and Opportunities

Despite the spectacular progress in flexible and transient CP systems, several challenges persist:

- Mechanical–electrical coupling: maintaining stable conductivity under repeated bending, stretching, and hydration cycles.
- Encapsulation and environmental stability: preventing water uptake or dopant leaching without sacrificing ionic accessibility.
- Biocompatibility and immune response: ensuring long-term tissue compatibility through surface modification or use of naturally derived matrices.
- Scalability and reproducibility: achieving uniform CP coatings and crosslinking over large areas for industrial-scale production.

Addressing these issues requires multidisciplinary optimization, merging polymer chemistry, biointerface engineering, and device architecture. Recent advances in 3D printing, inkjet patterning, and self-healing polymers promise to expand the design space further, enabling CP biosensors that are not only flexible and soft but also reconfigurable, self-repairing, and biodegradable [9,137].

### 7.5. Outlook: Toward Seamless Bioelectronic Integration

The convergence of CP-based electrochemical sensing with soft robotics, artificial skin, and implantable diagnostics heralds a new generation of bioelectronic systems that blur the boundary between living and artificial matter. PEDOT:PSS and its emerging derivatives—PEDOT:Tos, PEDOT:ILs, and P3CPT—will likely dominate this space, while hybridization with biopolymers and ILs will drive advances in long-term stability and environmental sustainability. In this context, CPs are evolving from passive conductors to active, adaptive materials capable of sensing, actuation, and communication within biological networks.

To move beyond proof-of-concept demonstrations, future CP-based electrochemical biosensors should increasingly be designed according to explicit material–device–application guidelines. At the materials level, polymer selection should balance mixed ionic–electronic conductivity with chemical stability in physiological media, favoring OMIECs or crosslinked PEDOT-based systems for long-term operation. At the device level, architectures that decouple signal transduction from irreversible faradaic reactions—such as impedance-based sensors or organic electrochemical transistors—are expected to offer improved stability and lower power consumption. At the interface level, biofunctionalization strategies should prioritize covalent or affinity-based immobilization combined with antifouling layers to minimize drift and inter-sample variability. The performance-envelope framework summarized in Table 2 provides a quantitative basis for translating these material–device–application guidelines into application-specific material selection and sensor architecture design, explicitly accounting for trade-offs between sensitivity, stability, and reproducibility.

Equally important is the adoption of standardized evaluation and reporting practices to enable meaningful comparison across laboratories and platforms. For wearable and implantable biosensors, this includes the use of defined calibration protocols, stable reference electrode strategies, and statistically validated performance metrics (e.g., LOD, linear range, stability retention, and reproducibility). Long-term testing under realistic mechanical deformation, hydration cycles, and biological matrices should become a minimum requirement rather than an optional characterization step.

From a translational perspective, realistic deployment of CP-based biosensors—particularly in wearable and implantable formats—will require convergence between materials engineering, device encapsulation, and regulatory-grade validation. For wearable systems, scalable printing or coating techniques combined with robust encapsulation and recalibration strategies are essential to ensure day-to-day reliability. For implantable devices, long-term biocompatibility, controlled degradation pathways, and predictable failure modes must be demonstrated through extended in vivo studies. In this context, CPs should be viewed not merely as functional sensing layers, but as integral components of bioelectronic systems whose lifecycle—from fabrication to operation and end-of-life—must be considered holistically.

The performance-envelope framework summarized in Table 2 provides a quantitative basis for translating these design guidelines into application-specific material and architecture choices.

## 8. Reliability, Drift, Fouling and Reproducibility

Despite remarkable advances in materials design and sensing performance, reliability remains the Achilles’ heel of CP-based electrochemical biosensors. In contrast to inorganic or carbon electrodes, CPs are soft, dynamic materials whose properties evolve over time due to redox cycling, hydration, and interaction with complex biological environments. Understanding and mitigating the sources of signal drift, biofouling, and inter-sample variability is therefore essential to transform laboratory prototypes into reproducible, clinically and industrially relevant devices.

**Figure 12:**
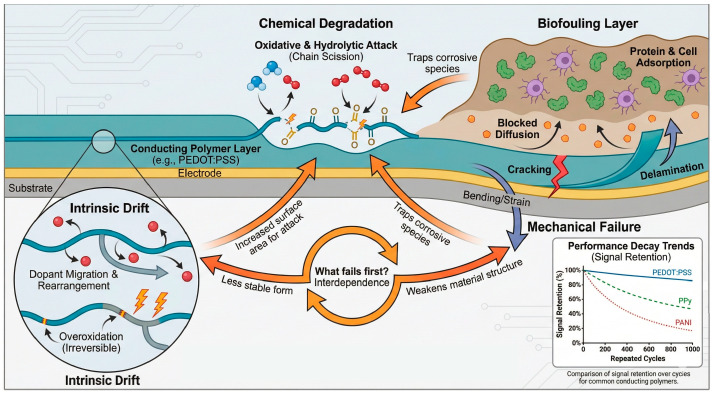
Degradation pathways and failure mechanisms in conducting polymer-based electrochemical sensors. The schematic illustrates the main degradation processes affecting conducting polymer (CP) sensing layers, including intrinsic drift due to dopant migration and overoxidation, chemical degradation driven by oxidative and hydrolytic attacks, biofouling-induced diffusion blocking, and mechanical failure under bending or strain. These mechanisms are interdependent and collectively contribute to signal drift, loss of sensitivity, and delamination over repeated operation cycles, as reflected by representative signal retention trends for common CPs.

As illustrated in Figure 12, reliability limitations in conducting polymer-based electrochemical sensors arise from the coupled and interdependent effects of intrinsic drift, chemical degradation, biofouling, and mechanical stress, which together govern signal stability, reproducibility, and long-term performance in aqueous and biological environments.

### 8.1. Electrical and Electrochemical Drift

Drift in CP-based sensors originates from intrinsic structural and chemical instabilities of the polymer matrix. Repeated redox cycling causes dopant redistribution, backbone oxidation, and conformational relaxation, altering conductivity and electrochemical response. For example, PEDOT:PSS films gradually lose conductivity upon overoxidation or dopant leaching, while PPy undergoes irreversible overoxidation after exposure to electrode potentials of E_SCE_ > 0.8 V in various neutral and acidic electrolyte solutions, leading to chain scission and decreased redox capacity [138].

The presence of mobile counter-ions (e.g., PSS^−^ and Cl^−^) facilitates ionic drift and water-layer-induced reorganization in PEDOT:PSS, particularly under continuous operation. These processes result in severe baseline shifts in open-circuit potential, often exceeding 5–10% within a few hours [139].

Mitigation strategies include:

- Crosslinking or encapsulation to immobilize dopants and limit swelling (e.g., GOPS-treated PEDOT:PSS).
- Operation within the polymer’s stable potential window (avoiding overoxidation).
- Periodic reconditioning cycles (e.g., potential pulsing to restore redox balance).
- Use of redox mediators to stabilize electron-transfer pathways without excessive polymer polarization.

Recent work also highlights the value of statistical drift modeling, applying machine learning to distinguish slow baseline variations from true analytical signals [140].

### 8.2. Chemical and Environmental Degradation

Exposure to oxygen, humidity, and reactive species induces chemical degradation of conjugated polymer thin films, particularly those with large interfacial areas. Photo-oxidative attack leads to the formation of carbonyl and sulfone/sulfoxide defects along the conjugated backbone, as directly evidenced by FTIR and XPS, disrupting π-delocalization and charge transport [141]. For PANI and PPy, hydrolysis of dopant acids or overoxidation reduces doping efficiency and conductivity. Thermal cycling or UV exposure accelerates these processes, causing irreversible changes in color and electrochemical signature [142,143].

Encapsulation within biopolymer or elastomer matrices (e.g., chitosan, polyurethane) can limit oxygen diffusion and enhance environmental robustness [76,144]. In implantable and wearable systems, repeated bending and hydration–dehydration cycles introduce mechanical failure modes such as cracking and delamination. Recent strategies based on elastic binders, ILs, and self-healing additives effectively suppress these effects while preserving mixed ionic–electronic transport [145,146].

### 8.3. Biofouling and Matrix Effects

Biofouling is arguably the most pervasive reliability issue in CP-based biosensors operating in biological fluids. Proteins, lipids, and cells readily adsorb onto the polymer surface, forming insulating layers that alter charge transfer and ion diffusion [147]. Even hydrophilic PEDOT:PSS, while less prone to fouling than metals, experiences progressive impedance increase and response suppression after prolonged exposure to serum or sweat [23].

Mitigation strategies include hydrophilic and zwitterionic coatings that form hydration shells resisting adsorption, dynamic antifouling approaches based on electrochemical cleaning pulses, and material-level design strategies [148]. Despite promising results, no universal antifouling solution exists, and performance strongly depends on the target matrix and testing conditions, highlighting the need for standardized evaluations under realistic flow and ionic environments [148].

### 8.4. Reproducibility and Inter-Batch Variability

Another obstacle to widespread adoption of CP-based sensors is poor reproducibility. Electropolymerization and film casting are highly sensitive to monomer purity, dopant concentration, temperature, and electrode geometry. Variations in film thickness or morphology can cause significant differences in R_ct_ and sensitivity among nominally identical devices. Statistical studies reveal that even within the same batch, signal dispersion can reach 10–20%, undermining calibration reliability. Automation of deposition (e.g., inkjet printing, electrodeposition under feedback control) and rigorous materials characterization (UV–Vis, XPS, AFM, profilometry) are now considered indispensable for reproducibility assurance. Furthermore, CP-based biosensors often lack reference standards—materials or protocols defining performance benchmarks—making cross-laboratory comparison difficult [149].

The field is gradually embracing metrological best practices, including:

- Reporting of sample size (n ≥ 3–5 devices per condition).
- Statistical treatment of calibration data (mean ± SD).
- Documentation of preconditioning and storage protocols.
- Use of reference electrodes and control experiments in complex matrices.

### 8.5. Coupled Degradation Mechanisms

In real applications, electrical, chemical, and biological degradations seldom occur independently. For instance, dopant leaching increases porosity and promotes biofouling; biofouling, in turn, restricts ion diffusion, leading to overoxidation during redox cycling.

Such feedback loops accelerate performance decay, producing nonlinear drift that complicates calibration and interpretation [9,76,150]. A deep understanding of these coupled mechanisms, supported by in situ monitoring (e.g., impedance spectroscopy over time, Raman or XPS tracking), is essential for designing stable CP interfaces [151,152,153].

### 8.6. Toward Standardized Reliability Testing

Addressing reliability requires not only better materials but also harmonized evaluation methodologies.

A minimum reliability protocol should include:

- Accelerated aging (continuous bias or redox cycling over 24–72 h).
- Repeated mechanical stress (bending, stretching cycles).
- Storage stability under defined humidity and temperature.
- Real-matrix exposure tests (serum, sweat, environmental water).

Reporting these parameters alongside electroanalytical metrics (LOD, linear range) would enable fair benchmarking and facilitate industrial translation.

## 9. Metrology, Reporting Standards and Benchmarking

As CP-based electrochemical biosensors transition from laboratory demonstrations to clinical and industrial applications, standardized metrology and transparent reporting become imperative. The extraordinary tunability of CPs—while scientifically attractive—introduces variability that can obscure real performance trends. Without consistent measurement protocols and unified metrics, comparisons across publications remain largely qualitative. This section outlines key principles and proposes a benchmarking framework for reproducible, quantitative assessment of CP-based (bio)sensors.

**Figure 13:**
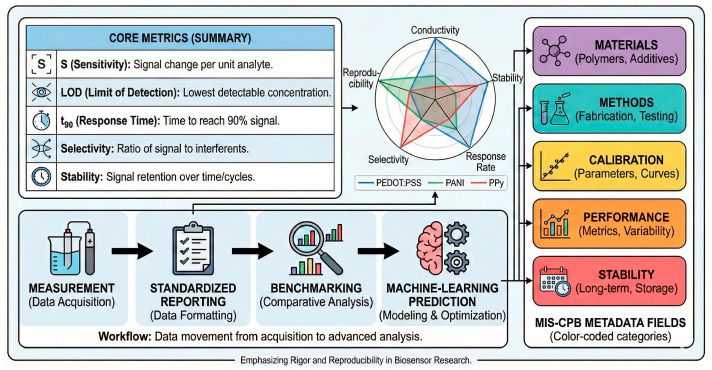
Benchmarking framework and standardized metrics for conducting polymer-based electrochemical (bio)sensors. The schematic summarizes core performance metrics—sensitivity, limit of detection, response time, selectivity, and stability—and outlines a standardized workflow from data acquisition and reporting to comparative benchmarking and machine-learning–assisted analysis. The framework highlights how materials selection, fabrication methods, calibration strategies, and metadata organization collectively govern reproducibility, cross-study comparability, and long-term performance evaluation of conducting polymer-based sensing platforms.

To address current limitations in cross-study comparability, Figure 13 outlines a standardized metrology and benchmarking framework for conducting polymer-based electrochemical sensors, linking core performance metrics with data reporting, comparative analysis, and predictive modeling.

### 9.1. The Metrological Gap

Most reports on CP-based sensors focus on relative improvements—e.g., “twofold sensitivity increase” or “lower LOD”—without clearly defining calibration methodology or uncertainty.

Discrepancies arise from variations in:

- Electrode area normalization (geometric vs. electroactive surface).
- Unit reporting (e.g., mA·mM^−1^·cm^−2^ vs. µA·µM^−1^·cm^−2^).
- LOD estimation, often inconsistently defined as 3σ/S or visual extrapolation.
- Reference electrode and potential scale, especially in miniaturized or wearable systems.
- Matrix composition, with artificial buffers replacing complex biological fluids.

These inconsistencies hinder the establishment of universal performance benchmarks and complicate meta-analyses. A “metrological culture” is therefore needed, where measurement uncertainty, traceability, and statistical validation are considered integral parts of materials research [149,154].

### 9.2. Standardized Metrics for Electrochemical Biosensors

To ensure comparability, at least five quantitative metrics should be reported in all CP-based sensor studies (Table 3). Additional descriptors include linear range, repeatability (RSD), reproducibility (inter-batch RSD), and biocompatibility (cytotoxicity or cell viability) for implantable systems. Reporting these values with mean ± standard deviation (n ≥ 3 devices) should become standard practice.

### 9.3. Reference Electrodes and Calibration Protocols

Reliable calibration requires stable reference and counter electrodes. For CP-based miniaturized or wearable devices, Ag/AgCl pseudo-references often exhibit potential drift of tens of millivolts over time [155,156]. Possible mitigation strategies include encapsulated Ag/AgCl inks or solid-state reference systems stabilized by ILs [155,156], periodic two-point recalibration, and the inclusion of internal redox references (e.g., ferrocene derivatives) embedded within the CP matrix for self-referencing [157].

Calibration curves should span at least one order of magnitude below and above the target concentration range, with regression statistics explicitly reported. For potentiometric sensors, the expected Nernstian slope (≈59 mV/dec) provides a straightforward self-consistency check of sensor accuracy [158].

### 9.4. Interlaboratory Reproducibility and Data Curation

Given the diversity of polymer formulations and testing conditions, interlaboratory reproducibility of CP-based sensors remains limited. Collaborative testing—where identical films or electrodes are distributed to multiple laboratories—has been shown to be effective in identifying systematic biases arising from fabrication, instrumentation, and sample handling [159]. To address these issues, recent perspectives emphasize the importance of open data repositories and standardized metadata to enable transparent cross-study benchmarking and meta-analysis [9]. The adoption of a Minimum Information Standard for CP-Based Biosensors (MIS-CPB), analogous to MIAME in genomics, would represent a practical first step toward reproducible, machine-readable datasets and data-driven performance assessment [9].

Core fields might include:

1. Polymer system and dopant.
2. Fabrication route and film thickness.
3. Electrochemical configuration (reference, counter, working).
4. Analyte and biological matrix.
5. Calibration methodology and statistics.
6. Stability and reproducibility data.

This standardized metadata would enable automatic comparison and machine-learning-driven analysis of CP sensor performance.

### 9.5. Benchmarking, Performance Envelopes, and Trade-Offs

**Figure 14:**
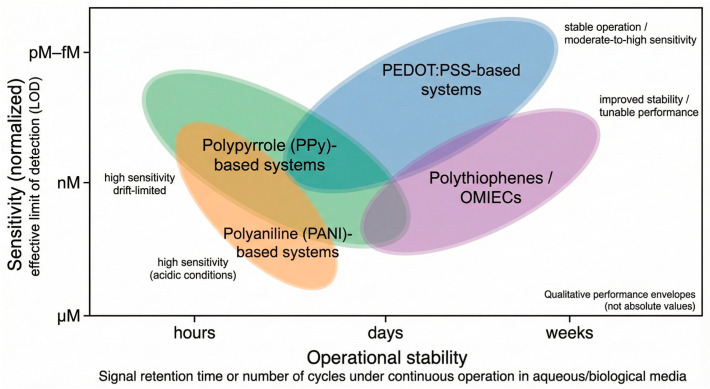
Qualitative performance envelopes of conducting polymer-based electrochemical sensing platforms. The diagram maps representative sensitivity versus operational stability for major conducting polymer families, including PEDOT:PSS-based systems, polypyrrole (PPy), polyaniline (PANI), and polythiophene/OMIEC materials. Shaded regions indicate qualitative performance envelopes derived from typical experimental ranges reported in the literature, highlighting inherent trade-offs between sensitivity, stability, and operating conditions rather than absolute record values. This representation enables comparative assessment of material platforms under realistic aqueous and biological sensing environments.

Direct quantitative comparison of CP-based electrochemical (bio)sensors across the literature is inherently challenging due to differences in device architecture, testing protocols, electrolyte composition, and metric definitions. For this reason, strict head-to-head ranking based on single reported values (e.g., the lowest limit of detection) is often misleading. Rather than compiling exhaustive lists of reported values, Table 2 consolidates representative order-of-magnitude metrics extracted from primary experimental studies, grouped by polymer family and sensing modality. This consolidation enables direct comparison of relative advantages and intrinsic trade-offs in terms of limit of detection, linear range, operational stability, and reproducibility under realistic operating conditions. Instead, a more meaningful critical assessment can be achieved by identifying performance envelopes (Figure 14)—that is, representative ranges of sensitivity, stability, and reproducibility that characterize specific polymer families and transduction modes under typical operating conditions. While previous reviews often report isolated record values or application-specific case studies, they rarely attempt a unified visualization of performance trade-offs across polymer families and sensing modalities. The performance-envelope approach adopted here is intended to explicitly address this gap by enabling cross-platform comparison under realistic operating constraints.

Across amperometric and voltammetric sensors, PEDOT:PSS-based systems frequently achieve lower effective detection limits and broader linear ranges, particularly when hybridized with catalytic nanomaterials. However, these advantages are often accompanied by sensitivity to hydration state and dopant redistribution, which can induce baseline drift over extended operation. In contrast, polypyrrole (PPy) and polyaniline (PANI) frequently exhibit higher intrinsic sensitivity toward redox-active analytes due to their rich redox chemistry and ease of enzyme entrapment, but often at the expense of long-term stability and reproducibility under neutral or physiological conditions. Emerging OMIECs and functionalized polythiophenes show improved stability in aqueous and biological environments, yet typically require more complex synthesis and processing routes.

Impedimetric and transistor-based platforms (e.g., organic electrochemical transistors) highlight a different trade-off landscape, where volumetric capacitance and ionic permeability enable ultra-low detection limits and label-free operation, but also amplify susceptibility to ionic drift, hydration fluctuations, and biofouling. As a result, stability metrics—such as signal retention over time or cycles—often become the dominant performance discriminant rather than sensitivity alone.

To visualize these trade-offs, Ashby-style performance maps plotting normalized sensitivity against operational stability provide an intuitive framework for materials selection. In such representations, PEDOT:PSS-based systems typically occupy a region characterized by moderate-to-high sensitivity combined with comparatively high stability, whereas PPy- and PANI-based systems cluster in regions of high sensitivity but reduced stability. Importantly, these maps reinforce that no single CP platform is universally optimal; rather, material choice must be guided by application-specific priorities, including acceptable drift, required lifetime, and deployment environment.

To address the lack of systematic quantitative comparison across conducting polymer-based biosensors, Table 2 consolidates representative performance envelopes—including electrical conductivity, limit of detection, sensitivity, selectivity, and operational stability—extracted from primary experimental studies and grouped by polymer family and sensing modality.

Rather than implying absolute ranking, the table highlights typical ranges, dominant failure modes, and best-fit use cases, thereby enabling readers to critically assess relative advantages and limitations across competing CP-based sensing platforms.

### 9.6. Toward Predictive and Data-Driven Metrology

Recent studies have demonstrated that machine learning models can successfully correlate polymer composition, dopant chemistry, and fabrication parameters with electrical conductivity or electrochemical response [160]. Extending this paradigm to biosensing could enable closed-loop materials optimization, where computational predictions guide polymer synthesis and device prototyping. However, the predictive power of such models ultimately depends on the quality, standardization, and reproducibility of the underlying data, underscoring the central role of robust metrology in data-driven materials research [161].

## 10. Emerging Directions and Future Outlook

Over four decades after the discovery of electronically CPs, their transformation into functional bioelectronic materials is reshaping electrochemical sensing and biosensing. PEDOT:PSS, PANI, PPy, and their derivatives have evolved from laboratory curiosities to core components of flexible, implantable, and transient devices. Yet the journey from proof-of-concept to reliable, manufacturable, and sustainable technology is far from complete. The next decade will see a shift from empirical optimization toward data-driven, biologically integrated, and environmentally conscious design of CP systems.

Bridging the gap between laboratory prototypes and real-world deployment will require not only continued materials innovation but also convergence toward standardized testing protocols, validated lifetime metrics, and reliability qualification pathways. For wearable and implantable biosensors in particular, marginal gains in analytical sensitivity are often less critical than reproducible performance under mechanical stress, stable reference electrode operation, resistance to biofouling, and predictable end-of-life behavior. Addressing these constraints demands an integrated approach in which polymer chemistry, device engineering, metrology, and regulatory considerations are treated as coupled design variables rather than sequential optimizations.

### 10.1. Beyond PEDOT:PSS: Next-Generation Mixed Conductors

PEDOT:PSS remains the benchmark CP; however, its dominance is increasingly challenged by next-generation OMIECs [16]. Unlike traditional doped systems, many OMIECs feature intrinsically charged backbones or zwitterionic side chains, eliminating the need for labile dopants and thereby reducing drift while enhancing volumetric capacitance and chemical stability in physiological media [16,162]. Their high ionic permeability and water stability make them promising candidates for long-term biosensing and neural interfacing [162]. Future research is expected to focus on side-chain engineering to tailor ionic selectivity, redox potentials, and bioreceptor compatibility, effectively bridging polymer chemistry and biointerface design [163].

### 10.2. Biohybrid and Living Polymer Interfaces

A major emerging direction lies in biohybrid materials, where CPs are combined with living or bioinspired components to create dynamic, self-regulating interfaces.

Recent breakthroughs include:

- Cell-laden CP hydrogels that can sense and respond to biochemical signals while supporting cell viability [164].
- Enzymatically polymerized CPs, using oxidoreductases to catalyze in situ polymer growth directly on biological substrates [165].
- Bacterial cellulose-based biodegradable composites, providing mechanically robust and flexible sensing platforms [166].
- Biomimetic self-assembly of CP nanofibers guided by peptides or polysaccharides to achieve hierarchical organization reminiscent of extracellular matrices [167].

These systems blur the boundary between living matter and synthetic conductors, paving the way toward adaptive bioelectronics capable of sensing, healing, or even metabolizing their surroundings.

### 10.3. Integration with Artificial Intelligence and Digital Twins

The increasing availability of standardized electrochemical data (Section 9) will fuel Artificial Intelligence (AI)-driven discovery and predictive modeling in CP-based sensing. Machine learning algorithms can already identify correlations between polymer structure, dopant chemistry, and sensor performance [160]. Future “digital twins” of CP sensors will simulate degradation, drift, and response under realistic conditions, enabling virtual prototyping before fabrication [168]. Coupling these models with high-throughput synthesis and automated testing could dramatically accelerate materials optimization [169]. Moreover, embedding lightweight AI algorithms directly into wearable CP biosensors will enable edge computing—real-time interpretation of biochemical data without cloud dependence, crucial for personalized health monitoring and low-power operation [170].

### 10.4. Transient, Sustainable, and Circular Bioelectronics

Sustainability is becoming a central criterion in materials design. Traditional CP fabrication often involves toxic solvents, heavy-metal catalysts, or non-biodegradable substrates. Emerging trends emphasize green synthesis (aqueous polymerization, bio-based monomers) and circular design, where devices are either recyclable or biodegradable [171,172].

Transient CP electronics, built on biopolymer substrates (cellulose, silk, chitosan) and doped with biodegradable ILs or natural acids, are attracting attention for environmental monitoring and resorbable implants [173]. Such devices address the growing demand for eco-compatible, disposable diagnostics while minimizing electronic waste. The integration of biodegradable energy sources—enzymatic biofuel cells or transient supercapacitors—will further expand the concept of fully self-contained green bioelectronics [174].

### 10.5. Toward Multifunctional and Multimodal Systems

The future of CP-based biosensors lies not in single-analyte detection but in multimodal sensing architectures that capture multiple physical and chemical cues simultaneously. CP composites that couple electrochemical sensing with mechanical, optical, or thermal transduction are emerging as truly multifunctional materials [175].

For instance, CP hydrogels doped with plasmonic nanoparticles can simultaneously detect biochemical changes (electrochemically) and temperature shifts (optically). Integration with piezoionic or triboelectric components enables energy harvesting and motion sensing within the same platform [176,177]. Such convergence will lead to closed-loop bioelectronic systems, capable of both sensing and actuation—for instance, detecting a metabolic imbalance and releasing a therapeutic dose in response.

### 10.6. Clinical Translation and Regulatory Pathways

For CP-based biosensors to reach the clinic, issues of standardization, biocompatibility, and long-term safety must be addressed. Regulatory frameworks currently used for medical devices are not yet adapted to dynamic, degradable polymers. Efforts are underway to establish ISO-like standards for soft and transient electronics, encompassing cytotoxicity, dissolution kinetics, and residual by-products [178].

Partnerships between academia, industry, and regulatory bodies will be essential to define testing protocols, shelf-life requirements, and data reliability criteria. Only through such interdisciplinary alignment will CP-based bioelectronics transition from curiosity to commodity.

### 10.7. The Grand Vision: Bio-Intelligent Materials

Ultimately, the evolution of CPs is steering toward bio-intelligent materials—platforms capable of sensing, processing, and adapting like living tissue. In this vision, CPs act not merely as passive electrodes but as dynamic, information-rich materials interfacing directly with biological systems. They may self-heal, self-adapt, or communicate with neural or biochemical networks in real time [179].

This convergence of soft matter physics, organic electronics, and synthetic biology represents one of the most compelling frontiers of materials science. In such a framework, the boundary between material and organism becomes increasingly porous—an outlook that resonates with the fundamental ethos of CPs: to conduct not only electricity, but life itself.

## 11. Conclusions and Design Guidelines

Over the past decade, CPs have evolved from niche functional coatings to core enabling materials for electrochemical sensing and biosensing.

Their combination of electronic conductivity, ionic permeability, and mechanical softness has redefined what an electrode can be—no longer a rigid transducer, but a living interface between chemistry, electronics, and biology.

From PEDOT:PSS thin films to PPy nanostructures and PANI composites, the field has expanded to encompass wearable, implantable, and even transient bioelectronic devices capable of operating within the complexity of biological environments.

However, the progress achieved is accompanied by persistent challenges—stability, drift, fouling, and lack of standardization—that must be addressed through systematic materials engineering and metrological rigor. The lessons gathered across the previous sections can now be distilled into a set of “Design Rules” for next-generation CP-based (bio)sensors.

### 11.1. Key Insights

1. Mixed conduction is both strength and weakness.
  The same ion–electron coupling that enables high sensitivity also introduces drift and environmental dependence. Engineering strategies must balance ionic mobility with structural stability via controlled doping and crosslinking.
2. Hierarchy matters.
  Nanoscale architecture (porosity, morphology) determines macroscopic performance. Hierarchical structures combining ordered conductive domains and soft, hydrated interfaces provide optimal performance for biological operation.
3. Interfaces define reliability.
  The polymer–biointerface, rather than the bulk material, dictates long-term reproducibility. Functionalization chemistry, antifouling coatings, and mechanical matching with tissues are as important as conductivity itself.
4. Metrology equals credibility.
  Reliable comparison requires standardized metrics (sensitivity, LOD, stability, selectivity) and transparent data reporting. Without reproducible benchmarks, no real progress can be measured.
5. Sustainability is not optional.
  As the field moves toward large-scale applications, the environmental and ethical footprint of CPs—solvent use, dopant toxicity, device disposal—will become critical design constraints.
6. Convergence defines the future.
  The fusion of CP materials with AI, biopolymers, and living systems will shape the coming decade of bioelectronics—where sensing, computation, and adaptation co-exist in a single material platform.

### 11.2. Design Rules for CP-Based Electrochemical Biosensors

As a synthesis of the concepts discussed throughout this review, the following section distills the core design criteria that govern the performance, stability, and translational potential of CP-based electrochemical biosensors. Rather than a simple checklist, these principles reflect an integrated framework—linking molecular design, interfacial engineering, processing, metrology, and sustainability—to guide the rational development of next-generation CP bioelectronics. The design of electrochemical biosensors based on CPs requires an integrated strategy in which polymer chemistry, film architecture, biofunctionalization, and measurement methodology collectively determine device performance.

A first consideration is polymer selection. PEDOT:PSS generally offers the best compromise between conductivity, stability, and processability, whereas PANI and PPy provide strong catalytic activity toward redox-active analytes. Functionalized PThs, in turn, exhibit improved stability at neutral pH and in physiological media. In all cases, CP selection should be guided by compatibility with the operating environment and by the redox potential of the target analyte. This is closely linked to the doping strategy, which is crucial for controlling mixed ionic–electronic conduction and long-term stability. Robust dopants such as PSS^−^ or TFSI^−^, sometimes combined with secondary dopants like EG or ILs, enhance conductivity while minimizing leaching, drift, and over-oxidation.

Morphological engineering is another key factor. Templating, self-assembly, and hierarchical porosity increase the electroactive surface area and facilitate ion transport, thereby improving sensitivity, response time, and mechanical robustness under repeated cycling in aqueous media.

To achieve biological selectivity, CPs are frequently functionalized with enzymes, antibodies, or biomimetic receptors. Immobilization strategies may rely on covalent coupling, affinity interactions, or mild crosslinking, always ensuring adequate hydration to preserve biorecognition activity. At the same time, reliable operation in complex matrices requires dedicated antifouling and stability measures. Hydrophilic or zwitterionic coatings, as well as the incorporation of biopolymers (e.g., cellulose, chitosan, natural hydrogels), reduce nonspecific adsorption and surface degradation, thereby mitigating signal loss over time.

From a metrological standpoint, reporting parameters such as sensitivity, LOD, t_90_, selectivity, and stability with clear statistics (e.g., mean ± SD, n ≥ 3) is essential. Such rigor enables reproducibility, transparent benchmarking, and meaningful cross-study comparisons.

Processing considerations are also becoming increasingly important. Aqueous or green synthesis routes, together with the avoidance of toxic solvents or catalysts, support sustainability and enhance biocompatibility for wearable or implantable applications. Integration concerns the coupling of CPs with flexible substrates, printed circuitry, and low-voltage architectures such as OECTs, enabling conformal, wearable, and implantable operation under dynamic mechanical conditions. The emergence of data-driven optimization further accelerates materials discovery. Machine-learning models trained on standardized datasets can correlate composition, morphology, and fabrication parameters with device performance, supporting rational and predictive design. Finally, modern CP-based biosensors should be conceived within a lifecycle design framework. Incorporating biodegradable or recyclable elements, enabling controlled disassembly or dissolution, and minimizing electronic waste aligns these technologies with the principles of sustainable and circular bioelectronics.

### 11.3. The Path Forward

The next generation of CP-based biosensors will not be defined by incremental improvements in sensitivity, but by robustness, integration, and intelligence. Materials will be selected not only for performance but also for stability, sustainability, and data interoperability. Success will depend on cross-disciplinary collaboration—polymer chemists developing stable, green OMIECs; bioengineers optimizing tissue interfaces; metrologists defining standards; and data scientists extracting predictive insights.

If the 2010s were the decade of discovery and diversification, the 2020s–2030s will be the era of convergence and reliability. By uniting design rules, standardized metrics, and sustainable chemistry, CPs are poised to underpin a new generation of adaptive, intelligent, and environmentally responsible bioelectronics—materials that conduct not just charge, but understanding.

For real-world deployment of CP-based electrochemical biosensors, stability and reproducibility must be treated as primary design constraints rather than secondary optimizations following sensitivity maximization. Design strategies should therefore prioritize (i) robust dopant systems and crosslinking to suppress drift, (ii) controlled morphology that balances ionic accessibility with mechanical integrity, (iii) explicit antifouling and encapsulation solutions tailored to the target matrix, and (iv) standardized calibration and reporting protocols that enable cross-study and cross-platform comparison. Only through such a reliability-first, metrology-driven approach can CP-based bioelectronic sensors transition from compelling laboratory demonstrations to scalable and clinically or industrially relevant technologies. Rather than proposing a single “best” conducting polymer for electrochemical biosensing, this review highlights that performance is governed by intrinsic trade-offs between sensitivity, stability, and reproducibility. By explicitly linking materials chemistry, processing routes, and metrological practices, we aim to provide a critical lens through which future studies can be evaluated and compared. This perspective is essential for transitioning CP-based sensors from proof-of-concept demonstrations to reliable, deployable bioelectronic systems.

## Figures and Tables

**Table 1:** Conductivity, water processability, flexibility modulus, stability and representative use for CP families.

CP Family	Typical σ (S cm^−1^)	Water Processable	Mechanical Compliance	Biocompatibility	Stability (Neutral pH)	Representative Use
PEDOT:PSS	10^2^–10^3^	✔	High	Excellent	Excellent	Neural, wearable, enzyme sensors
PANI (ES)	1–100	✖ (acidic only)	Low	Conditioned (pH dependent)	Poor–Moderate	Gas, pH, enzymatic sensors
PPy	1–50	✖ (Emulsion/Suspension Polymerization—Requires surfactants)	Low	Good (medium term)	Moderate	Enzyme entrapment, neurotransmitters
PTh/P3HT	10^−2^–10^1^	✖ Poor (with polar side chains)	Very low	Poor/Modest	Good	OECTs, printed sensors

Legend: ✔ indicates water processability; ✖ indicates lack of direct water processability (or only possible under restricted conditions).

**Table 3:** Quantitative metrics that should be reported in CP-based sensor studies.

Metric	Definition/Method	Notes/Common Pitfalls
Sensitivity (S)	S = ΔI/ΔC (amperometric) or ΔE/ΔlogC (potentiometric)	Always specify normalization (current per cm^2^, potential per decade) and calibration range.
Limit of Detection (LOD)	LOD = 3σ_blank/S	σ from ≥10 blank measurements; avoid visual estimation.
Response Time (t_90_)	Time to reach 90% of steady-state signal	Specify measurement conditions (stirring, temperature).
Selectivity	Ratio of target response to interferent response	Report interferent concentrations and experimental medium.
Stability/Retention (%)	(Signal after aging or cycles/initial signal) × 100	Specify number of cycles, time, and storage medium.

## Data Availability

No new data were created or analyzed in this study.
